# Identification of an extracellular vesicle-related gene signature in the prediction of pancreatic cancer clinical prognosis

**DOI:** 10.1042/BSR20201087

**Published:** 2020-12-04

**Authors:** Dafeng Xu, Yu Wang, Kailun Zhou, Jincai Wu, Zhensheng Zhang, Jiachao Zhang, Zhiwei Yu, Luzheng Liu, Xiangmei Liu, Bidan Li, Jinfang Zheng

**Affiliations:** 1 Department of Hepatobiliary and Pancreatic Surgery, Hainan General Hospital, Hainan Affiliated Hospital of Hainan Medical University, Haikou, Hainan, 570311, China; 2 Geriatrics Center, Hainan General Hospital, Hainan Affiliated Hospital of Hainan Medical University, Haikou, Hainan, 570311, China

**Keywords:** 3-PPI-Module, extracellular vesicle, ICGC, PAAD, TCGA, TME

## Abstract

Although extracellular vesicles (EVs) in body fluid have been considered to be ideal biomarkers for cancer diagnosis and prognosis, it is still difficult to distinguish EVs derived from tumor tissue and normal tissue. Therefore, the prognostic value of tumor-specific EVs was evaluated through related molecules in pancreatic tumor tissue. NA sequencing data of pancreatic adenocarcinoma (PAAD) were acquired from The Cancer Genome Atlas (TCGA) and International Cancer Genome Consortium (ICGC). EV-related genes in pancreatic cancer were obtained from exoRBase. Protein–protein interaction (PPI) network analysis was used to identify modules related to clinical stage. CIBERSORT was used to assess the abundance of immune and non-immune cells in the tumor microenvironment. A total of 12 PPI modules were identified, and the 3-PPI-MOD was identified based on the randomForest package. The genes of this model are involved in DNA damage and repair and cell membrane-related pathways. The independent external verification cohorts showed that the 3-PPI-MOD can significantly classify patient prognosis. Moreover, compared with the model constructed by pure gene expression, the 3-PPI-MOD showed better prognostic value. The expression of genes in the 3-PPI-MOD had a significant positive correlation with immune cells. Genes related to the hypoxia pathway were significantly enriched in the high-risk tumors predicted by the 3-PPI-MOD. External databases were used to verify the gene expression in the 3-PPI-MOD. The 3-PPI-MOD had satisfactory predictive performance and could be used as a prognostic predictive biomarker for pancreatic cancer.

## Introduction

Pancreatic cancer is currently one of the most malignant tumors in the world. There are as many as 400,000 deaths caused by pancreatic cancer each year worldwide with a 5-year survival rate of approximately 10% [1,2]. Due to the extremely low rate of early diagnosis, most patients have local infiltration or even distant metastasis when diagnosed [3]. Clinically, less than 20% of patients with pancreatic cancer can undergo surgical resection, and most patients will still eventually experience relapse and metastasis even after radical surgery [4]. The high mortality of pancreatic cancer results from two clinical dilemmas: the lack of effective early detection and the need for effective treatments [5]. Therefore, revealing its pathogenesis and seeking effective prognostic biomarkers and molecular targets are the core tasks for the treatment of pancreatic cancer.

Extracellular vesicles (EVs) are 40- to 100-nm vesicles with a membranous structure, and they are released by cells to regulate cell–cell communication by delivering functional molecules (such as proteins, nucleic acids, and lipids) to recipient cells [6]. Studies have shown that EVs are involved in many normal physiological processes, such as coagulation [7], autophagy [8], reproduction [9], and the nervous system [10]. EVs also play a role in pathological processes such as neurodegenerative diseases [11] and cancer [12]. In the tumor environment, EVs participate in a series of processes of tumorigenesis and progression, including inflammatory response, angiogenesis, cell proliferation, immunosuppression, epithelial–mesenchymal transition, and metastasis [13–16]. Therefore, EVs may be ideal biomarker candidates and therapeutic targets for anti-cancer therapy.

As the research focus of liquid biopsy, circulating EVs can be used as biomarkers for the early diagnosis of many tumors. Specifically, the contents of EVs (miRNA, protein, etc.) are promising diagnostic and prognostic biomarkers for tumors. The expression levels of miRN-196a and miR-1246 in the serum EVs of patients with early-stage pancreatic cancer are higher than in normal patients, and miRN-196a is more specific for pancreatic ductal adenocarcinoma, while miR-1246 is more specific for intraductal papillary mucinous carcinoma [17]. In addition, glypican-1(GPC1) in serum EVs is significantly increased in patients with pancreatic cancer, and even in patients with precancerous lesions, suggesting the great potential of GPC1 in EVs for the early diagnosis of pancreatic cancer [18]. However, EVs identified in body fluids are released from a mix of tumor tissue and other tissue. It is of great significance to differentiate tumor- specific EVs for the diagnosis and treatment of tumors. The protein content in EVs represents the proteomes of their origin cells [19,20]. Previous study indicate that circ-IARS was up-regulated both in exosome and pancreatic cancer cells to promote tumor metastasis [21]. In additional, Glypican-1 has been widely confirmed to be up-regulated in pancreatic cancer tissues and exosomes, which can be used as a diagnostic marker for pancreatic cancer [22–24]. These studies suggested the molecular similarity between exosomes and their original cells.

Therefore, the combined analysis of primary tumors and EVs would help to identify EVs as specific biomarkers for pancreatic cancer.

In the present study, the gene expression profile of protein-encoding genes in pancreatic cancer EVs were analyzed. Furthermore, through the protein–protein interaction (PPI) network method, an EV-related gene combination was established to predict pancreatic cancer prognosis, and it was validated in two independent data sets. In addition, the relationship between this model and the tumor microenvironment was explored.

## Materials and methods

### Subjects and clinical characteristics

Pancreatic adenocarcinoma (PAAD) data from three databases were used. They were the PAAD transcript sequencing data from The Cancer Genome Atlas (TCGA) (TCGA-PAAD), obtained from https://www.cancer.gov/about-nci/organization/ccg/research/structural-genomics/tcga, and the PAAD transcript sequencing data of the Australian cohort and the Canadian cohort from the International Cancer Genome Consortium (ICGC) (ICGC-PAAD-AU and ICGC-PAAD-CA, respectively), obtained from https://dcc.icgc.org/. These three independent cohorts covered two different quantification methods for transcripts and different populations in Asia, Europe, and the United States. The TCGA-PAAD data set contained 177 cases, and after excluding cases with an overall survival (OS) <30 days and without complete clinical information, the final sample size was 167. Similarly, ICGC-PAAD-AU data set contained 89 cases, and the final sample size was 89. The total number of cases and available samples in the ICGC-PAAD-CA data set were 165 and 159, respectively. Sample information is shown in Table 1 and Supplementary Table S1

**Table 1:** Clinical characteristics in three datasets of PAAD

	Training		Validation			
variables	TCGA-PAAD		ICGC-PAAD-AU		ICGC-PAAD-CA	
Age						
<60 ys	51	30.72%	22	24.72%	53	32.12%
≥60 ys	116	69.28%	67	75.28%	112	67.88%
Gender						
Female	76	45.51%	43	48.31%	77	46.67%
Male	91	54.49%	46	51.69%	88	53.33%
Stage						
I	18	10.78%	–	–	64	38.79%
II	142	85.02%	–	–	87	52.73%
III	3	1.80%	–	–	7	4.24%
Iv	4	2.40%	–	–	1	0.60%
Unknown	–	–	–	–	6	3.64%

### ExoRBase database

Pancreatic cancer-related EV genes were obtained from exoRBase (http://www.exorbase.org/exoRBase/browse/tomRNAIndex). The database contained 17,061 EV genes related to pancreatic cancer, and EV expression genes closely associated with pancreatic cancer were further enriched by subsequent bioinformatics analysis.

### Processing of gene expression data

The TCGA-PAAD data set contained two sets of data: read counts and fragments per kilobase million (FPKM); the ICGC-PAAD-AU and ICGC-PAAD-CA data sets were read counts. The FPKM data of the TCGA-PAAD, ICGC-PAAD-AU, and ICGC-PAAD-CA data sets were all Z-transformed for standardization. The read counts data of the TCGA-PAAD data set were used in the differential expression analysis of normal para-cancerous tissue and tumor samples.

EBSeq software [25] was used to analyze the gene expression differences between the tumor and adjacent normal samples in the TCGA-PAAD cohort (Supplementary Table S2), and 415 specific differentially expressed genes were obtained according to posterior probability of differential expression (PPDE) >0.95. An intersection operation was further performed on these differentially expressed genes and PAAD-specific EV genes from the exoRBase database, and 287 PAAD EV-specific genes were identified (Supplementary Table S3).

### PPI processing

Based on the 287 genes specifically expressed in PAAD EVs, STRING (https://string-db.org) was used to construct the PPI network with an interaction score ≥0.4. The PPI network, which is shown in Supplementary Figure S1, contained 205 nodes and 484 interactions. Based on this PPI network, MCODE was used to identify PPI modules. The degree cutoff was 2, the node score cutoff was 0.2, the *K*-score was 2, and the Max.depth was 100. Finally, 12 potential PPI modules were identified (Supplementary Figure S2).

### Identification of PPI modules related to clinical stage

To further identify modules with significant predictive performance, integration of these potential PPI modules with the TCGA-PAAD expression profile was performed. First, the expression score of each module (expression score, *e*) was calculated. In a given module M with m genes, the expression score *e* of M in sample j was defined as [26]:

$$e_{j}=\sum_{i}^{m}\frac{Z_{ij}}{\sqrt{m}}$$

Here, *Z_ij_* is the *z*-transformed gene expression value of gene i. Then, the discriminant score of the M module S(M) was defined as the mutual information (MI) between e′ and the clinical stage class (*c*):

$$\text{S}(\text{M})=\text{MI}(e^{\prime },c)=\overset{e^{\prime }}{\underset{X}{\sum }}\overset{c}{\underset{Y}{\sum }}p(X,Y)\log\frac{p(X,Y)}{p(X)\left(X\right)P(Y)}$$

Here, *e*′ is the discrete form of *e*. The expression score *e* was discretized to 9 (log_2_ (*N*) + 1), and *N* was the sample size. The calculation process is shown in Supplementary Figure S3. In terms of clinical classification, stages I and II were classified into the low stage group and stages III and IV were classified into the high stage group.

Subsequently, the same number of genes as in the M module were randomly extracted from the PPI network to calculate the MI value of the randomly selected ‘module’. Each module was performed 1000 times randomly. Statistical analysis was performed using the calculation results of random sampling and the actual module. A module with a significant *P*-value (*P*<0.001) was selected for subsequent construction of the signature (Supplementary Table S4).

### Development of the predictive prognosis signature

To construct a gene signature based on the PPI network, the gene expression scores of candidate modules (as defined above) were used. Based on the random forest (RF) algorithm, the R package *randomForest* was used for feature selection and construction of the signature. An initial RF of 5000 trees was used to estimate the predictive importance of each candidate module iteration. A stepwise backward selection method was used to determine the optimal combination of recursive prediction candidate modules. In each iteration, 10% of the features were excluded, and the remaining features were used to build an RF model containing 3000 trees [27,28]. The program stopped when there were only two functions left. Among all iteration results, the RF model with the fewest features was selected. Finally, three PPI modules met the requirements.

### Identification of the tumor microenvironment

We use the method of Cao et al. [23] to estimate the tumor microenvironment state. Hypoxic metabolites of different cancer types were obtained from previous studies [29]. Core angiogenic biomarkers of primary tumors were obtained from Masiero et al. [30]. Inflammatory cytokinins were used to estimate intratumoral inflammation levels [31]. The scores of hypoxia, angiogenesis, and inflammation in tumor cells were calculated by averaging the Z-normalized expression values of the corresponding biomarker genes. The abundance of immune and non-immune cells in the tumor microenvironment was calculated by CIBERSORT (http://cibersort.stanford.edu/) through the gene expression profile for tissue-infiltrating cells.

### Protein expression analysis of 3-PPI-MOD genes

The Human Protein Atlas (HPA) provides tissue and cellular distribution information of 26,000 human proteins, mainly using specific antibodies to study the expression of proteins in cell lines, normal tissue, and tumor tissue. We explored 15 genes (CNR2, CX3CR1, CXCR5, GNGT1, GPR18, GPSM2, NMU, NPY1R, SSTR5, GIP, NTS, LEP, H3F3C, HIST1H2BC, and HIST2H3C) in normal pancreatic tissue and cancerous pancreatic tissue.

### mRNA expression validation in the external cohort

The expression of the 15 genes in cancerous pancreatic tissue and normal pancreatic tissue was analyzed in GSE71989 [32] and GSE15471 [33], and a box diagram was drawn in terms of genetic units.

### Mutation analysis

cBioPortal integrates genomic data, including somatic mutations, DNA copy-number alterations, mRNA and microRNA (miRNA) expression, DNA methylation, protein enrichment, and phosphorylated protein enrichment. It was used to perform mutation correlation analysis of the 15 genes (OncoPrint and histogram display of gene mutation) in the Pancreas (ICGC), Pancreas (QCMG2016), Pancreas (TCGA PanCan2018), Pancreas (TCGA), and Pancreas (UTSW) data sets of the cBioPortal database.

### Analysis of 3-PPI-MOD genes in the exoRBase database

The expression data of 3-PPI-MOD genes in normal (NP), coronary heart disease (CHD), colorectal cancer (CRC), hepatocellular carcinoma (HCC), pancreatic cancer (PAAD), and breast cancer (BC) were obtained from the exoRBase database [34].

### Statistical methods

The R package *clusterProfiler* [35] was used for Gene Ontology (GO) and Kyoto Encyclopedia of Genes and Genomes (KEGG) pathway enrichment analysis of the PPI modules. Gene set enrichment analysis (GSEA) was used to compare the gene sets of interest and the subgroups of patients classified by 3-PPI-MOD [36]. The correlations between PPI modules and hypoxia, angiogenesis, and inflammation scores as well as stromal cell abundance were evaluated by Pearson correlation. The Benjamini–Hochberg method was used to adjust multiple tests through the false discovery rate (FDR). The Kaplan–Meier curve and log-rank test were used to compare the survival rates of patients in the low- and high-risk groups allocated by the 3-PPI-MOD. A multivariate Cox model was used to assess the 3-PPI-MOD signature’s predictive performance for prognosis. All statistical analyses were performed using R software (version 3.3.1). A *P*-value <0.05 was considered significant.

## Results

### Identification of PPI modules related to PAAD

**Figure 1:**
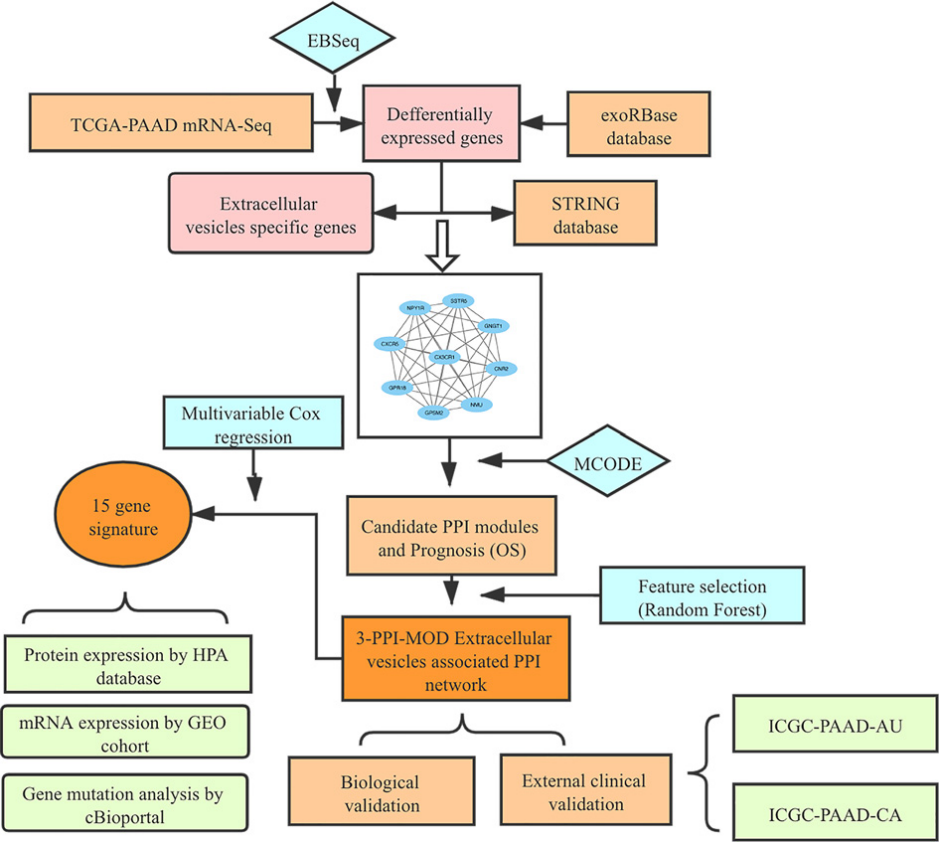
The Study Workflow The PPI network of extracellular vesicle proteins isolated from PAAD was integrated with the gene expression profile of the training data set. Candidate modules with locally maximal relapse scores were identified by a greedy searching approach. The random forest algorithm was used to establish a network-based signature for relapse risk. The 3-PPI-MOD signature was further validated for prognosis in 2 independent data sets. Biological validation was performed by comparing the signature with various databases. Abbreviations: PPI, protein–protein interaction; 3-PPI-MOD, 3 PPI modules.

Figure 1 depicts the overall flow chart of the present study. For the specific proteins in the EVs of pancreatic cancer, 205 proteins are located on the reference PPI network. The EV-related PPI network was integrated with the gene expression profile of the training cohort. Next, we identified 12 PPI modules using the MCODE algorithm. The method of random sampling was used to estimate the significance of the score of each module. The results showed that the prognostic discriminant scores of the 12 modules were significantly higher than those of accident score (*P*<0.001; Supplementary Table S4). The details of the 12 candidate modules are shown in Supplementary Figure S2.

**Figure 2:**
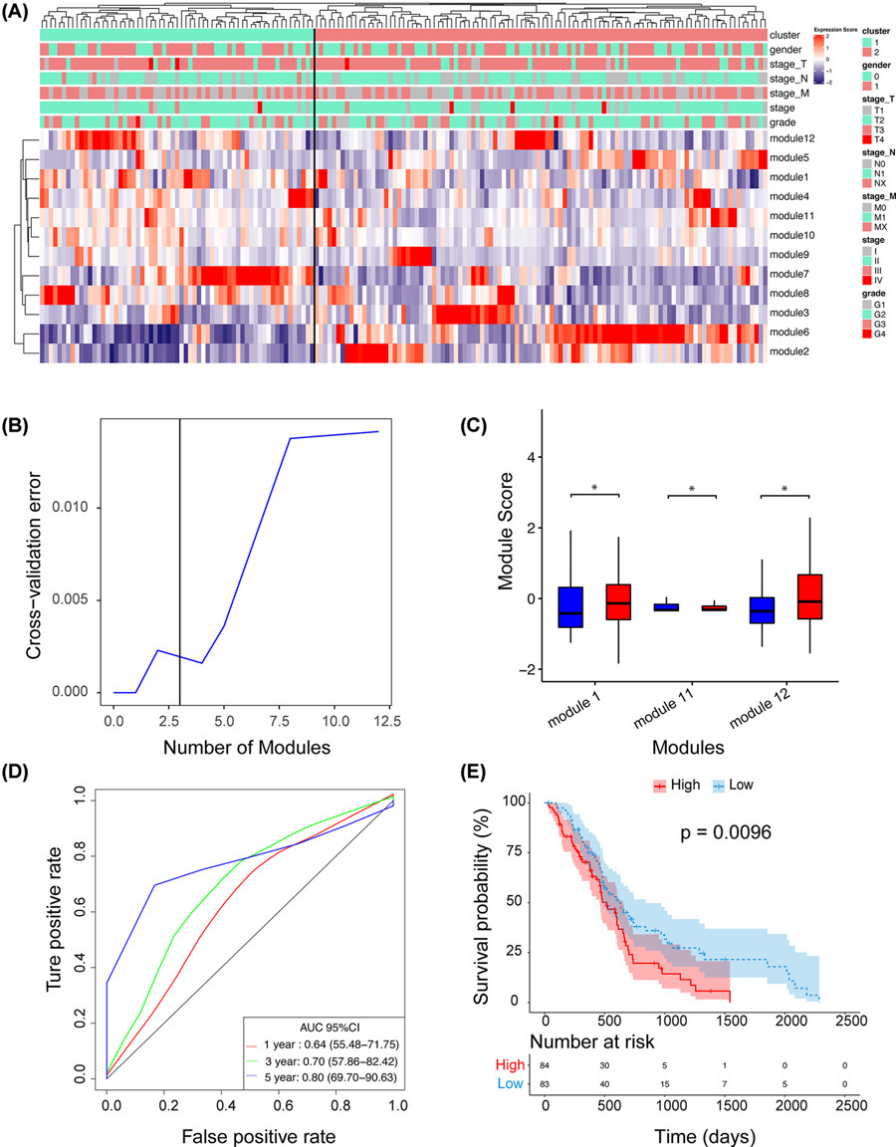
Establishing a Prognostic Signature Based on an Extracellular Vesicle-Associated Network (**A**) Expression score profiles of candidate PPI modules. The heatmap shows the expression scores of the 12 PPI modules. Unsupervised hierarchical clustering analysis was performed for modules (rows) and samples (columns). The upper panel indicates the clinicopathological variables of the tumor samples. (**B**) Feature selection based on the random forest algorithm. The *x*-axis indicates the number of used variables (modules). The *y*-axis is the cross-validation error of each prediction model. (**C**) Expression scores of the three modules selected in (B). The *P*-value was calculated by *t* test. (**D**) The recurrence risk predicted by the 3-PPI-MOD was used to perform ROC analysis. AUROC is presented. (**E**) Survival analysis for the 3-PPI-MOD in all patients. The Kaplan–Meier curve and log-rank test were used to compare overall survival between the different risk subgroups. * means P-value <0.05.

The expression score heat map of the 12 PPI modules is shown in Figure 2A. Through the unsupervised cluster algorithm, all modules were divided into two categories. Patients were also divided into two main subgroups. These two subgroups were significantly correlated with clinical stage (adjusted *P*=0.033, chi-square test) and grade (adjusted *P*=0.046, chi-square test) (Figure 2A), but not with other clinicopathological factors such as age, sex, or tumor, node, metastasis (TNM) stage.

### Establishment of a predictive signature for cancer recurrence risk

An optimal model for predicting prognosis classification was established using the RF algorithm for the 12 PPI modules. We found that the verification error decreased when it was greater than the 2-PPI-MOD and increased after the 4-PPI-MOD. The optimal error interval was between 2 and 4 (Figure 2B).

As seen in Supplementary Figure S4, starting from the 4-PPI-MOD, the importance suddenly decreased. The importance curve was arranged from high to low, and there was a significant derivative mutation, with the 3-PPI-MOD as the local maximum. We found that module 1 and module 12 in the 3-PPI-MOD were up-regulated in tumors, and module 11 was down-regulated in tumors (Figure 2C). The 1-, 3-, and 5-year risk were predicted by the receiver operating characteristic (ROC) curve, and the results showed that the 1-year area under the curve (AUC) was 0.64, the 3-year AUC was 0.70, and the 5-year AUC was 0.8, indicating that the 3-PPI-MOD had good 5-year performance (Figure 2D). Taking the median of the predicted risk coefficient as the boundary value, the patients were divided into the high- and low-risk groups. The Kaplan–Meier curve confirmed that the 3-PPI-MOD could predict the OS rate of patients (*P*=0.0096; Figure 2E). Multivariate Cox regression showed that the 3-PPI-MOD was an independent prognostic factor of OS (adjusted hazard ratio [HR] = 2.0265; 95% CI, 1.19–3.43; *P*=0.00873; Supplementary Table S5).

### Functional enrichment analysis of the genes in the modules

**Figure 3:**
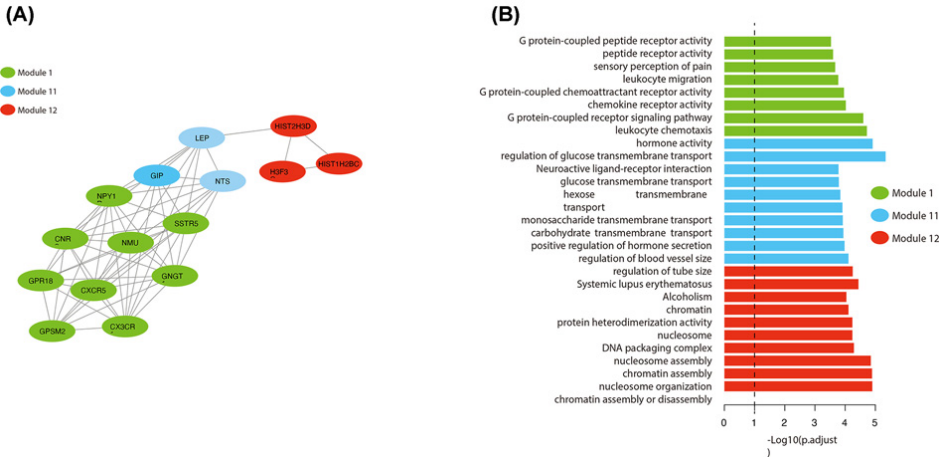
Biological Characteristics of the 3-PPI-MOD Signature (**A**) View of interactions of the 3-PPI-MOD signature. Three modules are shown by different colors. (**B**) Enrichment analysis for the 3 modules. The significant GO terms or KEGG pathways are listed on the left. Bars indicate the enrichment significance (log10-transformed p.adjust). The color legend indicates different modules.

In this 3-PPI-MOD signature, 9, 3, and 3 genes were contained in mod_1, mod_11, and mod_12, respectively. Module 11 was connected to Module 1 and Module 12 in the PPI network (Figure 3A). Pathway enrichment analysis showed that mod_1 was associated with G protein-coupled receptor activation, mod_11 was significantly associated with cell membrane-related pathways, and mod_12 was widely involved in DNA damage and repair (Figure 3B).

### Validation of the 3-PPI-MOD signature in independent cohorts

**Figure 4:**
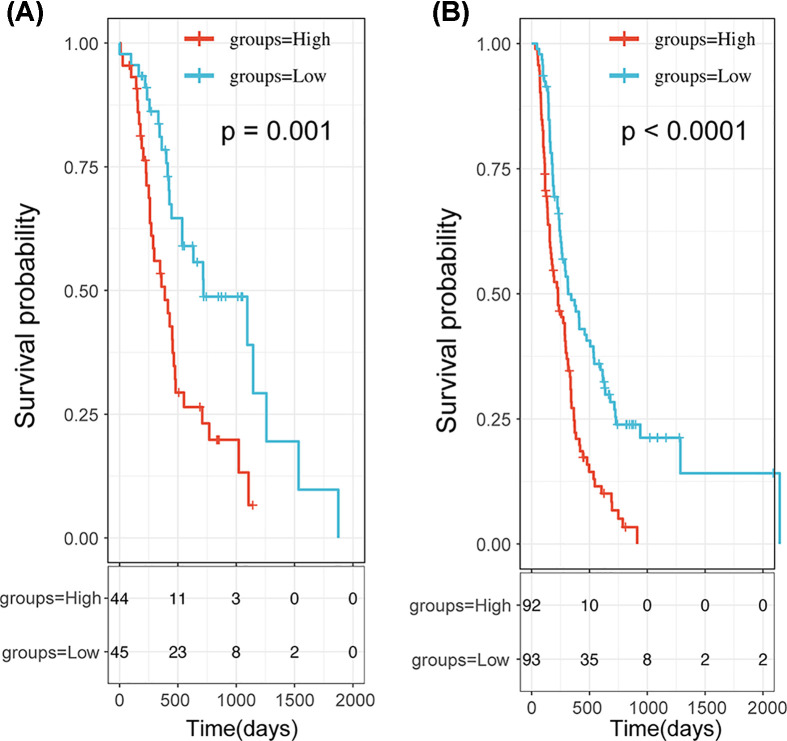
Survival Analysis of the 3-PPI-MOD Signature in 2 Validation Data Sets In each data set, patients were predicted to be in low- or high-risk subgroups by the signature. The Kaplan–Meier curve and log-rank test were used to compare the overall survival of patients in different risk subgroups. (**A**) The survival curve of 3-PPI-MOD in ICGC-PAAD-AU. (**B**) The survival curve of 3-PPI-MOD in ICGC-PAAD-CA

Next, we validated the prognosis of the 3-PPI-MOD in two validation data sets. The Riskscore distribution of 3-PPI-MOD in ICGC-PAAD-AU were presented in Supplementary Figure S5A. The Kaplan–Meier curve showed that high-risk patients in 3-PPI-MOD had worse prognoses than low-risk patients in ICGC-PAAD-AU (Figure 4A, logrank *P*=0.00049). The Riskscore distribution of 3-PPI-MOD in ICGC-PAAD-CA were shown in Supplementary Figure S5B, and Kaplan–Meier curve showed that high-risk patients in 3-PPI-MOD also had worse prognoses than low-risk patients in ICGC-PAAD-CA (Figure 4B, logrank *P*<0.0001).

### The EV-related signature showed better predictive performance on prognosis than gene expression

**Figure 5:**
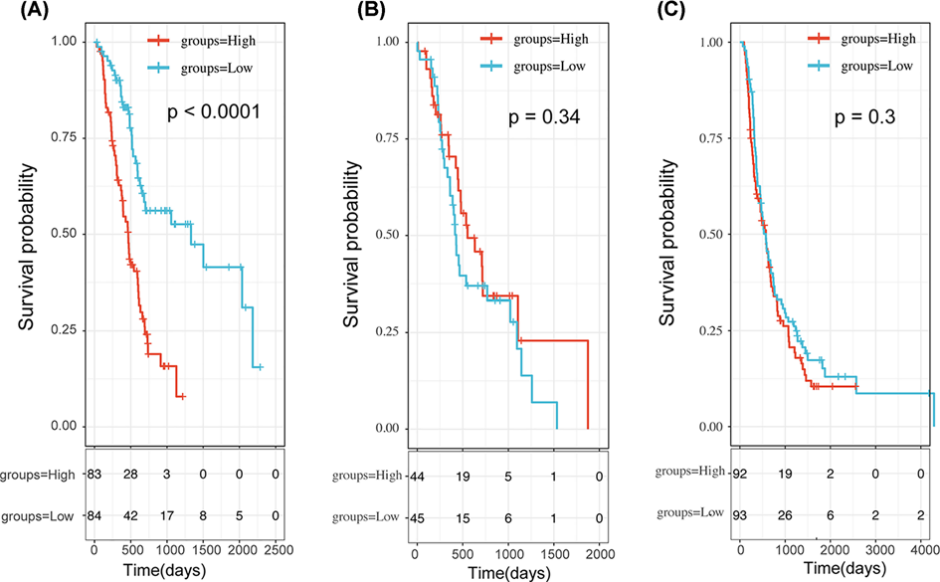
Predictive value of the 15-Gene Signature Survival analysis of the 15-gene signature in three data sets. In each data set, patients were predicted to be in low- or high-risk subgroups by the signature. The Kaplan–Meier curve and log-rank test were used to compare the overall survival of patients in different risk subgroups: TCGA-PAAD (**A**) ICGC-PAAD-AU (**B**) and ICGC-PAAD-CA (**C**).

The EV-related 3-PPI-MOD was compared with the 15-gene signature (constructed only based on gene expression). Multivariable Cox regression was used to calculate the risk scores of the 15-gene signature, using the median value as the threshold to divide the high- and low-risk groups. In the TCGA cohort, the 15-gene signature could significantly distinguish the prognosis of patients (Figure 5A), but this was not significant in the validation cohorts (Figure 5B,C). This shows that compared with the signature constructed by pure gene expression, the 3-PPI-MOD showed better prognostic value.

### The 3-PPI-MOD reflected tumor interstitial interaction and the hypoxic tumor microenvironment

**Figure 6:**
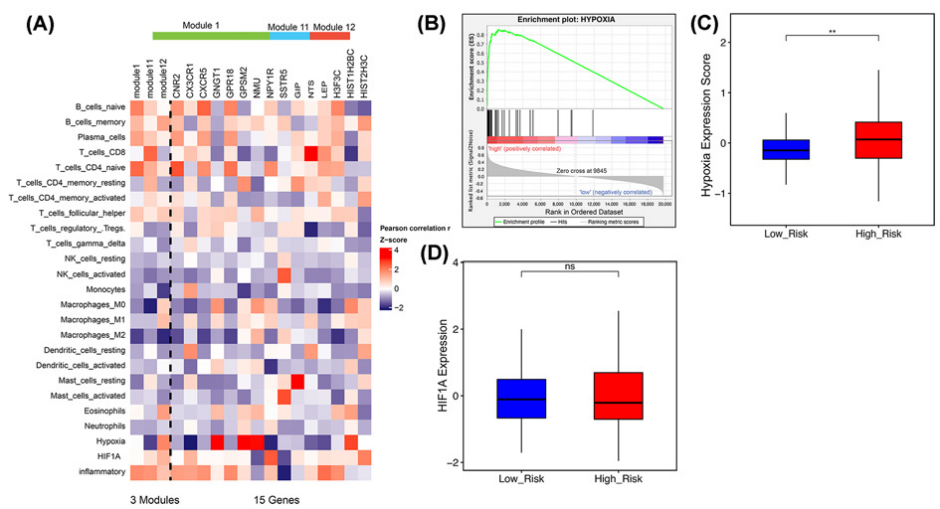
The 3-PPI-MOD Reflects Interactions Between Cancer Cells and the Tumor Microenvironment (**A**) Correlation analysis of the signature with stromal cell abundance and microenvironment status. The abundance of immune cells was calculated by CIBERSORT. The microenvironment status for hypoxia, angiogenesis, and inflammation were estimated as described in the ‘Materials and Methods’ section. (**B**) GSEA of the hypoxia metagene against the risk subgroups classified by the 3-PPI-MOD. (**C**) The boxplot shows intra-tumoral hypoxia scores between the low- and high-risk subgroups. The *P*-value was calculated by *t* test. (**D**) The boxplot shows HIF1A expression levels between tumors with low and high risk. The *P*-value was calculated by *t* test. ** means P-value < 0.01.

Non-cancer cells in tumors play an important role in the construction of the tumor microenvironment, especially in terms of immune cell infiltration. It was speculated that the EV-specific 3-PPI-MOD might be related to the tumor microenvironment in the present study. Therefore, CIBERSORT was used to identify the proportion of cell subtypes associated with immunity in the TCGA data set. The expression of some genes in the 3-PPI-MOD had a significant positive correlation with the proportion of immune cells. For example, model 1 showed a significant positive correlation with CD4 T cells but a negative correlation with M2 macrophages, while model 11 showed a significant positive correlation with CD8 T cells and a negative correlation with M0 macrophages. Model 12 was negatively correlated with most immune cells and most closely related to M2 macrophages (Figure 6A).

Furthermore, we analyzed the relationship between the 3-PPI-MOD and tumor microenvironment. We found that mod_12 was positively correlated with hypoxia scores, while mod_1 and mod_11 were negatively correlated with hypoxia. Not only that, mod_12, mod_1, and mod_11 were related to inflammation and hypoxia inducible factor 1A (HIF1A) (Figure 6A). Genes related to the hypoxia pathway were significantly enriched in the high-risk tumors predicted by the 3-PPI-MOD (Figure 6B).

In addition, hypoxia expression scores were significantly upregulated in the high-risk subgroup identified by the 3-PPI-MOD (Figure 6C), but the expression level of HIF1A was not related to the clinical risk of the 3-PPI-MOD (Figure 6D).

### Validation of 3-PPI-MOD gene expression in protein, mRNA, and EVs

In the 3-PPI-MOD, mod_1 contained 9 genes (GPR18, GPSM2, CXCR5, CX3CR1, CNR2, NMU, GNGT1, NPY1R, SSTR5), mod_11 contained 3 genes (GIP, NTS, LEP), and mod_12 contained 3 genes (H3F3C, HIST2H3C, HIST1H2BC).

**Figure 7:**
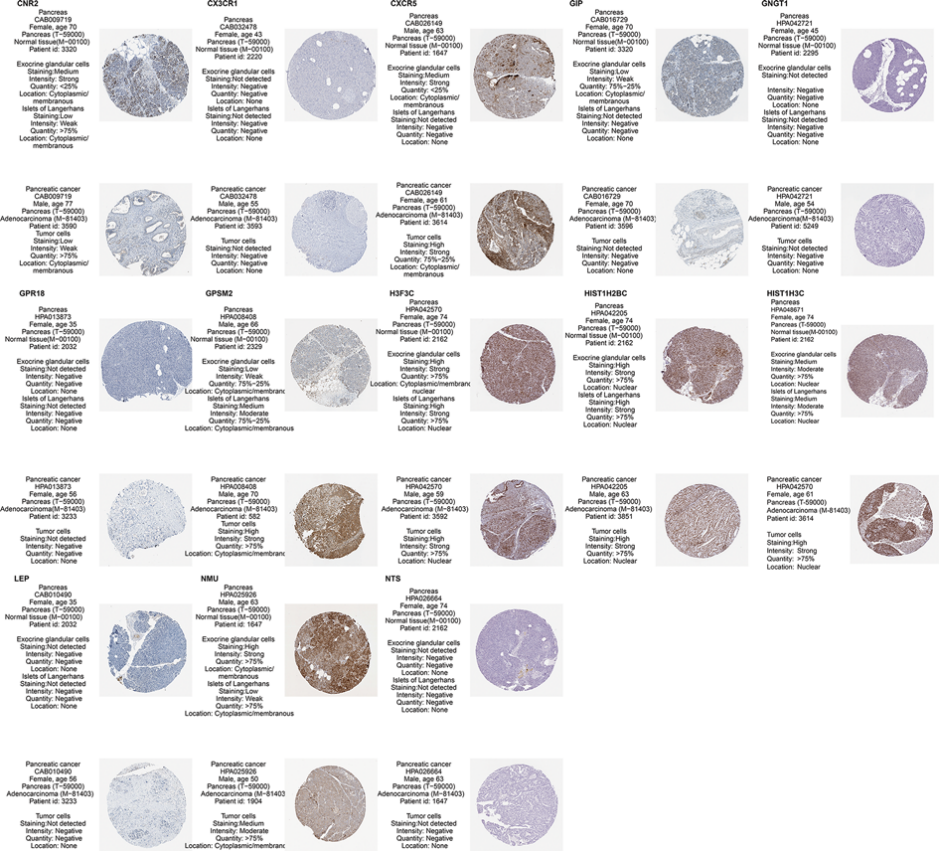
The protein expression of 15 genes was validated in The Human Protein Atlas database between tumors and normal controls

The results of protein expression analysis of the 15 genes showed that CX3CR1, GIP, GNGT1, GPR18, NTS, and LEP were negative in tumor tissue and normal tissue, the expression of CNR2 was significantly lower in tumor tissue, H3F3C and HIST1H2BC were not significantly different between tumor tissue and normal tissue, and CXCR5, HIST2H3C, and GPSM2 were more highly expressed in tumor tissue than in normal tissue. NMU was highly expressed in both tumor tissue and normal tissue, and NPY1R and SSTR5 were not collected in the HPA database (Figure 7).

**Figure 8:**
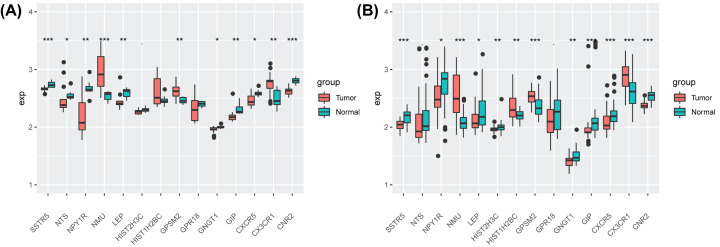
mRNA expression validation in the external cohort (**A**) The expression of the 15 genes in cancerous pancreatic tissue and normal pancreatic tissue was analyzed in GSE71989. (**B**) The expression of the 14 genes in cancerous pancreatic tissue and normal pancreatic tissue was analyzed in GSE15471

The expression of the 15 genes in GSE71989 (Figure 8A) and GSE15471 (Figure 8B) was analyzed(H3F3C were not found in both cohorts). Among them, the expression of CNR2, GIP, GNGT1, NPY1R, SSTR5, and LEP in tumor tissue was significantly lower than in normal tissue, while the expression of GPSM2, and NMU was significantly highly expressed in tumor tissue.

**Figure 9:**
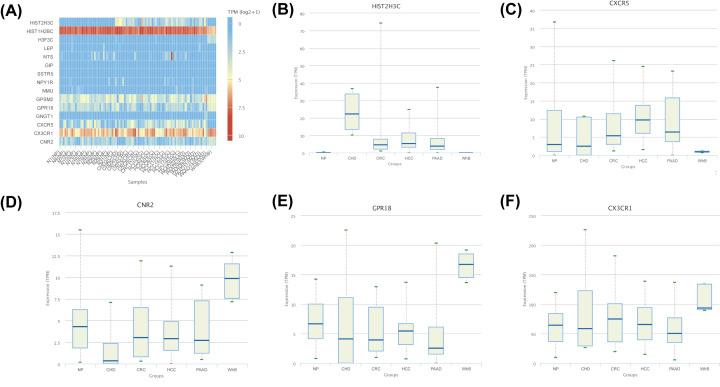
Analysis of 3-PPI-MOD genes in the exoRBase database (**A**)The expression of 3-PPI-MOD genes in normal (NP), coronary heart disease (CHD), colorectal cancer (CRC), hepatocellular carcinoma (HCC), pancreatic cancer (PAAD), and breast cancer (**B,C**). B-F. the box plot of gene expression of CXCR5, HIST2H3C, CNR2, CX3CR1 and GPR18 in normal (NP), coronary heart disease (CHD), colorectal cancer (CRC), hepatocellular carcinoma (HCC), pancreatic cancer (PAAD), and breast cancer (BC).

In the exoRBase database, we found that CXCR5 and HIST2H3C were more highly expressed in PAAD tissue than in normal tissue, while CNR2, CX3CR1, and GPR18 were lower in PAAD tissue than in normal tissue (Figure 9).

### Gene mutation analysis and correlation analysis with HIF1A

**Figure 10:**
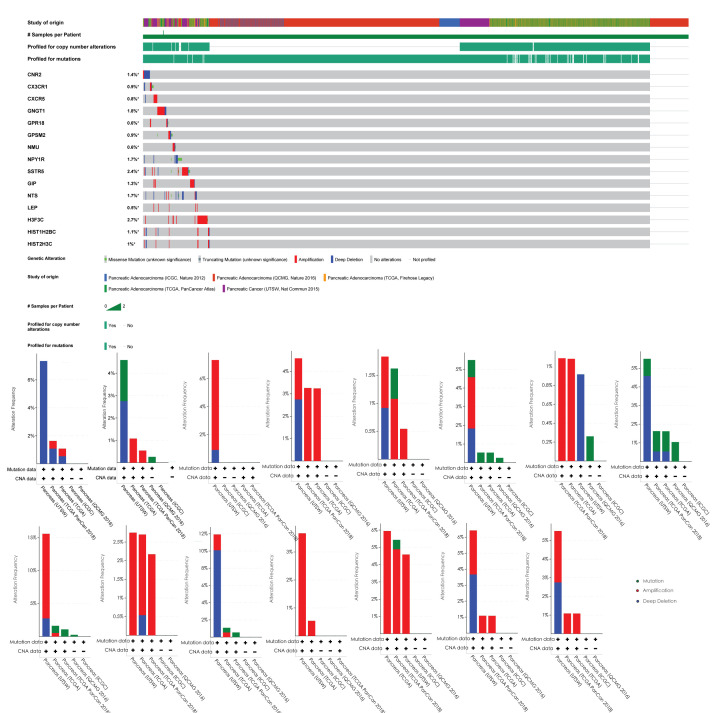
The gene mutation analysis The overall distribution of mutations of the 15 genes in the liver cancer data set in the cBioPortal database. The gene with the highest mutation rate was H3F3C, accounting for 2.7%, and the mutation type was amplification and point mutation. The mutation rate of the SSTR5 gene accounted for 2.4%, and the mutation type was mainly amplification

We analyzed the mutations of the 15 genes in the liver cancer data set in the cBioPortal database. The gene with the highest mutation rate was H3F3C, accounting for 2.7%, and the mutation type was amplification and point mutation. The mutation rate of the SSTR5 gene accounted for 2.4%, and the mutation type was mainly amplification (Figure 10).

## Discussion

*In vivo*, EVs are released in almost all types of cells and have been found in a variety of body fluids, including blood, urine, saliva, bile, and cerebrospinal fluid [37,38]. EVs contain various lipids [39], proteins [40], metabolites [41], mRNA fragments, non-coding RNAs [42], and even DNA fragments [43]. EVs isolated from the body fluids of patients with cancer contain cancer-related molecules, such as amplified oncogenes, oncoproteins, specific miRNAs, and mutant mRNA or DNA fragments [44,45]. Due to the interference of EVs from other tissues, it is still difficult to assess the role of tumor- specific EVs [46]. The molecular characteristics of EVs are consistent with their originating cells [47], showing the potential for combined analysis of primary tumor and EVs to distinguish tumor-specific EVs.

Although miRNA and non-coding RNAs play an important role in post-transcriptional regulation, gene transcripts are the direct vectors of transcriptional regulation, therefore, in this study we systematically analyzed the extracellular vesicle-related gene expression in pancreatic cancer and the relationship between prognosis. PPI network analysis is an important method for developing cancer biomarkers. Through PPI network analysis, the corresponding PPI modules were screened. The PPI modules identified from PAAD EVs characteristic expression genes were closely related to clinical stage, further confirming the potential clinical significance.

Many studies have shown the importance of EVs in tumor progression. In addition, EVs as biomarkers for monitoring the occurrence of cancer and tracking the progress of cancer have gained widespread attention from researchers [48]. Giampieri et al. compared EpCAM-positive EV levels in pancreatic ductal carcinoma patients (*n*=19) before and after chemotherapy, with the results showing that EpCAM-positive EV levels before chemotherapy were associated with shorter progression-free survival and overall survival, while the increase of EpCAM-EV was associated with better progression-free survival during chemotherapy [49]. In the present study, the PPI modules obtained were further analyzed using the RF algorithm, and the 3-PPI-MOD was generated. Kaplan–Meier analysis confirmed that this 3-PPI-MOD was closely related to survival. Further, multivariate Cox regression showed that the 3-PPI-MOD was an independent prognostic factor for OS. Functional enrichment analysis of the 3 modules revealed that mod_1 was associated with G protein-coupled receptor activation, mod_11 was significantly associated with cell membrane-related pathways, and mod_12 was widely involved in DNA damage and repair. These pathways play an important role in the malignant progression of PAAD [50,51], suggesting that this 3-PPI-MOD is closely related to the malignant phenotype of patients with PAAD. More importantly, the 3-PPI-MOD showed good predictive performance in 2 independent external validation sets: ICGC-PAAD-AU and ICGC-PAAD-CA. In addition, it performed better than a signature based on pure gene expression. In summary, the 3-PPI-MOD based on PAAD EVs genes had reliable prediction of prognosis in different populations and thus possesses great clinical significance.

The tumor microenvironment (TME) is composed of tumor cells and the stromal microenvironment [52]. Tumor cells, interstitial cells, and the extracellular matrix interact to produce and release various chemokines, cytokines, and other mediators, forming inflammatory states in tissues and the immunosuppressive TME and helping tumor cells escape the body’s immunity surveillance, which ultimately leads to tumorigenesis, tumor development, and metastasis [53,54]. Much research has shown the importance of EVs in the TME [55,56,57].

In the present study, we first discovered that some gene expression in 3-PPI-MOD was significantly positively correlated with the proportion of immune cells in the microenvironment. For example, model 1 showed a significant positive correlation with CD4 T cells but a negative correlation with M2 macrophages, while model 11 showed a significant positive correlation with CD8 T cells.

More importantly, genes related to the hypoxia pathway were significantly enriched in the high-risk tumors predicted by the 3-PPI-MOD, and hypoxia expression scores were significantly upregulated in the high-risk subgroup identified by the 3-PPI-MOD. These results indicated that the hypoxic microenvironment might be involved in the expression and prognosis of characteristic proteins of PAAD EVs, revealing the importance of the hypoxic microenvironment. However, no significant differences were found between 3-PPI-MOD and hypoxia-inducible factor 1(HIF1) expression in the high-and low-risk groups, so we hypothesize that hypoxia associated with 3-PPI-Mod is unlikely to be mediated by HIF1 and may be associated with abnormal expression of other genes in the hypoxia-related pathway.

In the 3-PPI-MOD, mod_1 contained 9 genes (GPR18, GPSM2, CXCR5, CX3CR1, CNR2, NMU, GNGT1, NPY1R, SSTR5), mod_11 contained 3 genes (GIP, NTS, LEP), and mod_12 contained 3 genes (H3F3C, HIST2H3C, HIST1H2BC).

GPR18 is a widely studied G protein-coupled receptor that is selectively expressed in immune cells as a cannabinoid receptor [58,59]. The role of GPR18 in tumors is not clear, and this study revealed its prognostic role in pancreatic cancer for the first time. The proteins encoded by GPSM2 belong to the protein family that regulates the activation of G proteins. GPSM2 plays the role of an oncogene in liver cancer [60], breast cancer [61], and pancreatic cancer [62]. In addition, GPSM2 can inhibit the proliferation and metastasis of lung cancer cells, and the specific mechanism is related to the AKT/ERK pathway [63,64]

CXCR5 belongs to the CXC chemokine receptor family, which binds to B lymphocyte chemokines and participates in B-cell migration. It is widely involved in the malignant progression of tumors and the abnormal activation of multiple signaling pathways in tumor cells [65,66]. The overexpression of CXCR5 in pancreatic cancer is a potential therapeutic target [67]. CX3CR1 is a chemokine that binds to CX3CL1 and mediates its adhesion and migration. However, the relationship between its expression and the patient prognosis s is not clear [68,69]. CNR2 encodes cannabinoid receptor protein, which is a member of the G protein-coupled receptor family and mediates the inhibition of adenylate cyclase. There are poor prognostic factors in solid tumors such as renal carcinoma [70], colorectal cancer [71], and breast cancer [72,73], but their role in pancreatic cancer is still unknown. Polypeptides encoded by NUM play an important role in pain, stress, and immune-mediated inflammatory diseases [74]. In tumors, their expression is increased in liver cancer and endometrial cancer [75,76].

NPY1R encodes neuropeptides that are widely expressed in the central nervous system and function through G protein-coupled receptors and participate in biological processes such as food intake and regulation of circadian rhythms [77]. NPY1R is a poor prognostic factor for prostate cancer and melanoma [78,79], but its role in pancreatic cancer is not clear. As a somatostatin receptor, SSTR5 plays a variety of biological roles on normal and tumor tissue targets by interacting with somatostatin [80]. Genetic variation in SSTR is closely related to pancreatic cancer risk [81]. GIP, a member of the glucagon superfamily, is a powerful insulin secretion stimulant that plays an important role in maintaining glucose homeostasis [82].

LEP encodes proteins secreted by white adipocytes into circulation and plays a major role in regulating energy homeostasis. Some researchers identified the expression of LEP in mouse serum exosomes; in breast cancer, LEP enhances intercellular signal communication by promoting exocrine secretion [83]. LEP is also involved in the regulation of pancreatic cancer cell proliferation, energy metabolism, and chemotherapy resistance [84–87].

NTS encodes a common precursor of neuropeptide M and neurotensin, and it plays an important role in the central nervous system; it has an important role in tumors, enhancing the progression of pancreatic cancer, prostate cancer, lung cancer, breast cancer, and colon cancer [88,89]. In addition, GNGT, H3F3CP, HIST2H3C and HIST1H2BC have not been reported to be related to the prognosis of tumor patients, and it was found for the first time in this study that HIST2H3C and HIST2H3C can be used as prognostic markers of pancreatic cancer.

Although the present study was based on large-sample omics data, and a prognostic model based on EV-specific genes was constructed, there were still some limitations. The conclusions in the present study were mainly based on bioinformatics analysis, so further validation in clinical samples is still needed. In addition, samples involved in the present study were all from retrospective studies, so further studies are necessary for clinical applications.

In summary, the prognostic model (3-PPI-MOD) based on pancreatic cancer extracellular vesicle characteristic proteins in the present study showed great value for clinical prognosis. In addition, the 3-PPI-MOD was closely related to the hypoxic microenvironment of tumors. The present study provides new ideas for assessing the prognostic value of EV-related molecules in tumor tissue, as well as for the prognosis of patients with pancreatic cancer.

## Supplementary Material

Supplementary Figures S1-S5 and Tables S1-S5

## Data Availability

The data sets used and/or analyzed during the current study are available from the corresponding author on reasonable request

## Abbreviations

EV: extracellular vesicle
PAAD: pancreatic adenocarcinoma
PPI: protein–protein interaction
TCGA: The Cancer Genome Atlas
TME: tumor microenvironment
