# Supplementary material for: Identification of an extracellular vesicle-related gene signature in the prediction of pancreatic cancer clinical prognosis
Source: Biosci Rep. 2020 Dec 4;40(12):BSR20201087. doi: 10.1042/BSR20201087 (PMC7724614; doi:10.1042/BSR20201087)
Supplement: Supplementary Figures S1-S5 and Tables S1-S5 [file BSR-2020-1087_supp.pdf]

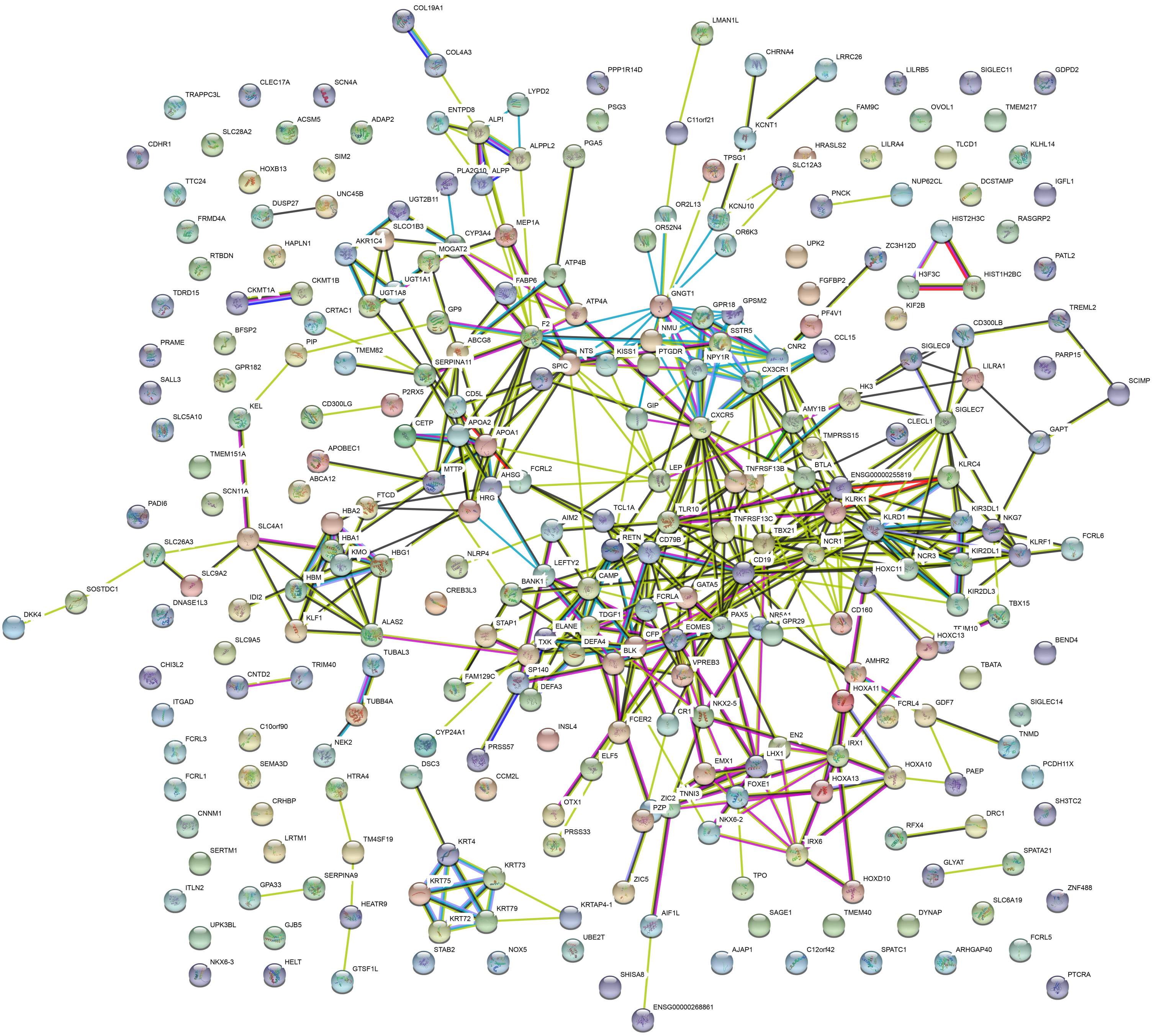
COL19A1
LMAN1L
COL4A3
CHRNA4
LRRC26
PPP1R14D
LILRB5
SIGLEC11
GDPD2
TRAPPC3L
CLEC17A
SCN4A
LYPD2
ENTPD8
PSG3
FAM9C
OVOL1
TMEM217
ACSM5
ADAP2
ALPI
C11orf21
KCNT1
CDHR1
SLC28A2
ALPPL2
PGA5
HRASLS2
LILRA4
TLCD1
KLHL14
TPSG1
SIM2
PLA2G10
ALPP
SLC12A3
HOXB13
TTC24
UNC45B
UGT2B11
OR2L13
PNCK
NUP62CL
DCSTAMP
IGFL1
DUSP27
KCNJ10
FRMD4A
SLCO1B3
CYP3A4
MEP1A
OR52N4
OR6K3
HIST2H3C
RASGRP2
AKR1C4
UPK2
HAPLN1
MOGAT2
ZC3H12D
RTBDN
ATP4B
H3F3C
HIST1H2BC
PATL2
UGT1A1
CKMT1B
FABP6
TDRD15
CKMT1A
UGT1A8
GNGT1
FGFBP2
ATP4A
GPR18
GPSM2
KIF2B
CRTAC1
GP9
PF4V1
TREML2
F2
CD300LB
BFSP2
NMU
ABCG8
SSTR5
PIP
SIGLEC9
PRAME
TMEM82
NTS
KISS1
CCL15
PTGDR
CNR2
SERPINA11
NPY1R
PARP15
SPIC
CX3CR1
SALL3
GPR182
LILRA1
SCIMP
HK3
P2RX5
GIP
CD5L
AMY1B
KEL
CD300LG
SIGLEC7
SLC5A10
CXCR5
CETP
APOA2
CLECL1
GAPT
APOA1
TMPRSS15
TMEM151A
APOBEC1
BTLA
MTTP
LEP
KLRC4
TNFRSF13B
ENSG00000255819
FCRL2
ABCA12
AHSG
SCN11A
KLRK1
FTCD
HRG
PADI6
TCL1A
TLR10
KIR3DL1
HBA2
KLRD1
NKG7
AIM2
TNFRSF13C
SLC4A1
RETN
TBX21
FCRL6
HBA1
CD79B
NCR1
KLRF1
SLC26A3
KMO
HBG1
KIR2DL1
NLRP4
CD19
NCR3
SLC9A2
SOSTDC1
LEFTY2
HBM
HOXC11
CAMP
BANK1
GATA5
KIR2DL3
IDI2
CREB3L3
FCRLA
DKK4
DNASE1L3
CD160
KLF1
TDGF1
PAX5
NR5A1
TBX15
STAP1
GPR29
ALAS2
ELANE
EOMES
TRIM10
SLC9A5
CFP
BEND4
TXK
DEFA4
HOXC13
TUBAL3
BLK
CHI3L2
TRIM40
VPREB3
AMHR2
CNTD2
FAM129C
SP140
TBATA
DEFA3
HOXA11
ITGAD
TUBB4A
FCRL4
GDF7
SIGLEC14
NKX2-5
CR1
FCER2
C10orf90
INSL4
CYP24A1
FCRL3
EN2
TNMD
NEK2
PRSS57
IRX1
LHX1
SEMA3D
ELF5
EMX1
HOXA10
PCDH11X
FCRL1
HTRA4
DSC3
CCM2L
PAEP
FOXE1
HOXA13
OTX1
TNNI3
CRHBP
ZIC2
SH3TC2
CNNM1
PZP
DRC1
NKX6-2
RFX4
LRTM1
PRSS33
TM4SF19
IRX6
KRT4
SERTM1
SPATA21
GLYAT
KRT73
SERPINA9
TPO
ZIC5
HOXD10
KRT75
GPA33
ITLN2
ZNF488
HEATR9
SLC6A19
KRT72
KRT79
KRTAP4-1
AIF1L
SAGE1
TMEM40
DYNAP
UPK3BL
GJB5
FCRL5
UBE2T
STAB2
NOX5
AJAP1
C12orf42
SPATC1
ARHGAP40
NKX6-3
HELT
GTSF1L
SHISA8
PTCRA
ENSG00000268861

Figure S1. The PPI network based on the 287 genes specifically expressed in PAADEVs

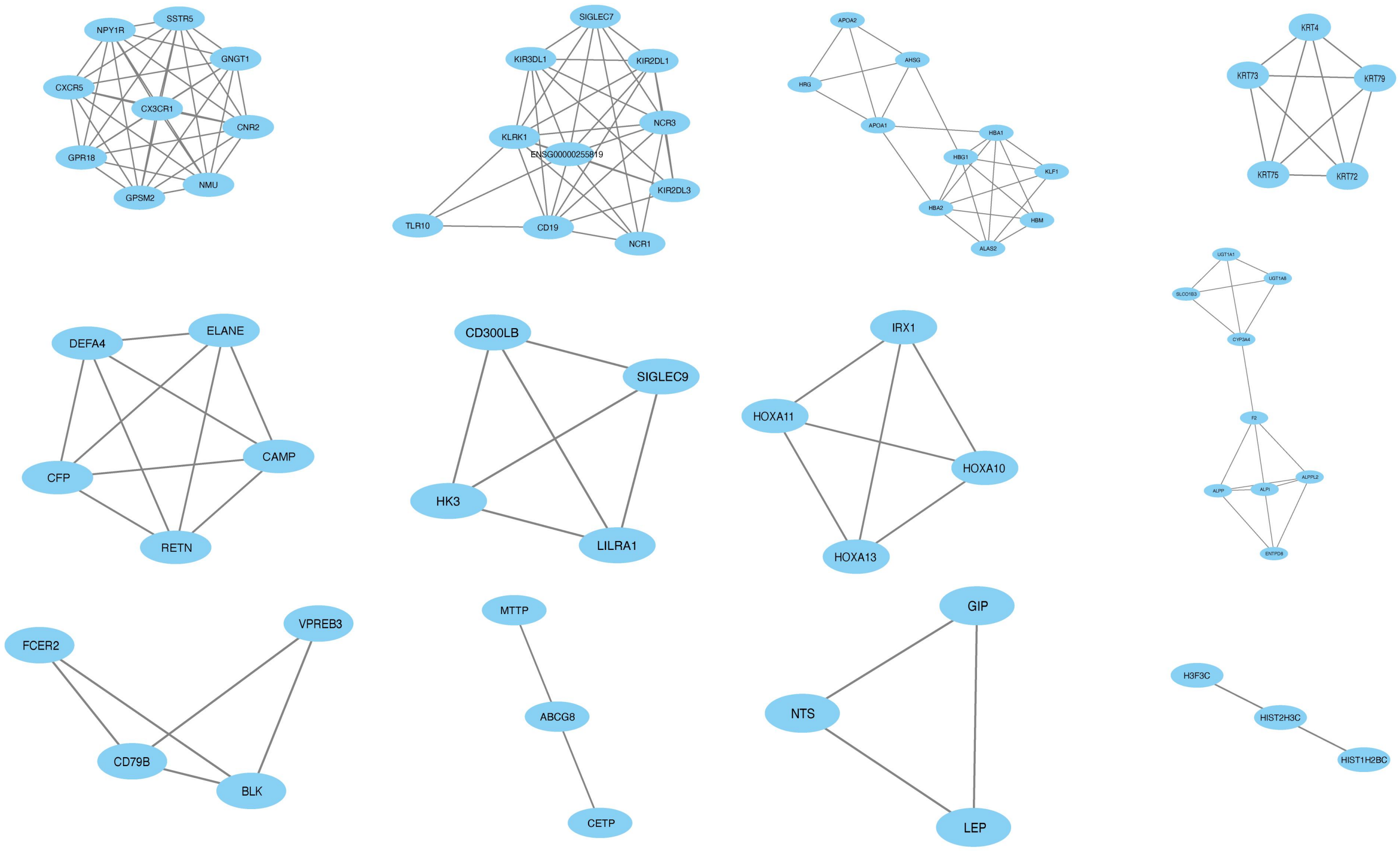

SSTR5
NPY1R
GNGT1
CXCR5
CX3CR1
CNR2
GPR18
NMU
GPSM2
SIGLEC7
KIR3DL1
KIR2DL1
NCR3
KLRK1
ENSG00000255819
KIR2DL3
TLR10
CD19
NCR1
APOA2
AHSG
HRG
APOA1
HBA1
HBG1
KLF1
HBA2
HBM
ALAS2
KRT4
KRT73
KRT79
KRT75
KRT72
UGT1A1
UGT1A8
SLCO1B3
CYP3A4
F2
ALPPL2
ALPP
ALPI
ENTPD8
DEFA4
ELANE
CAMP
CFP
RETN
CD300LB
SIGLEC9
HK3
LILRA1
IRX1
HOXA11
HOXA10
HOXA13
FCER2
VPREB3
CD79B
BLK
MTTP
ABCG8
CETP
GIP
NTS
LEP
H3F3C
HIST2H3C
HIST1H2BC

Figure S2. 12 potential identified PPI modules

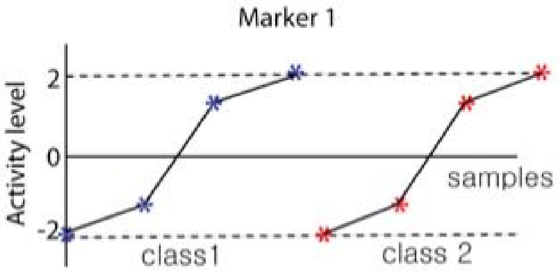

Original activity $a = [\ -2 \quad -1 \quad 1 \quad 2 \quad -2 \quad -1 \quad 1 \quad 2\ ]$

Discretized activity $x = [\ -1.65 \quad -0.55 \quad 0.55 \quad 1.65 \quad -1.65 \quad -0.55 \quad 0.55 \quad 1.65\ ]$

Class labels $c = [\ 1 \quad 1 \quad 1 \quad 1 \quad 2 \quad 2 \quad 2 \quad 2\ ]$

Joint probability

c \ x	-1.65	-0.55	0.55	1.65	
1	1/8	1/8	1/8	1/8	1/2
2	1/8	1/8	1/8	1/8	1/2
	1/4	1/4	1/4	1/4	

$$MI(\mathrm{a}',\mathrm{c}) = \sum_{x\in \mathrm{a}'}\sum_{y\in \mathrm{c}} p(x,y)\log\frac{p(x,y)}{p(x)p(y)} = \left(\frac{1}{8}\times\log\left(\frac{1}{8}/\left(\frac{1}{4}\times\frac{1}{2}\right)\right)\right)\times 8 = 0,$$ p-value = 1

t-score(a′, c) = 0, p-value = 1

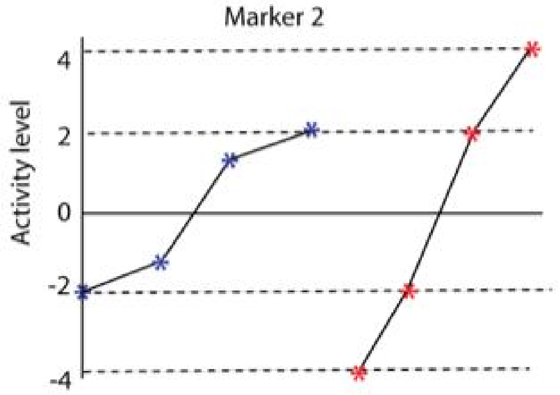

Original activity $a = [\ -2 \quad -1 \quad 1 \quad 2 \quad -4 \quad -2 \quad 2 \quad 4\ ]$

Discretized activity $x = [\ -1.15 \quad -1.15 \quad 1.15 \quad 1.15 \quad -3.45 \quad -1.15 \quad 1.15 \quad 3.45\ ]$

Class labels $c = [\ 1 \quad 1 \quad 1 \quad 1 \quad 2 \quad 2 \quad 2 \quad 2\ ]$

Joint probability

c \ x	-3.45	-1.15	1.15	3.45	
1	0	1/4	1/4	0	1/2
2	1/8	1/8	1/8	1/8	1/2
	1/8	3/8	3/8	1/8	

$$MI(\mathrm{a}',\mathrm{c}) = \sum_{x\in \mathrm{a}'}\sum_{y\in \mathrm{c}} p(x,y)\log\frac{p(x,y)}{p(x)p(y)} = \left(\frac{1}{4}\times\log\left(\frac{1}{4}/\left(\frac{3}{8}\times\frac{1}{2}\right)\right)\right)\times 2 + \left(\frac{1}{8}\times\log\left(\frac{1}{8}/\left(\frac{1}{8}\times\frac{1}{2}\right)\right)\right)\times 2 + \left(\frac{1}{8}\times\log\left(\frac{1}{8}/\left(\frac{3}{8}\times\frac{1}{2}\right)\right)\right)\times 2 = 0.2157$$

p-value = 0

t-score(a′, c) = 0, p-value = 1

Figure S3. Identification of PPI modules related to clinical stage

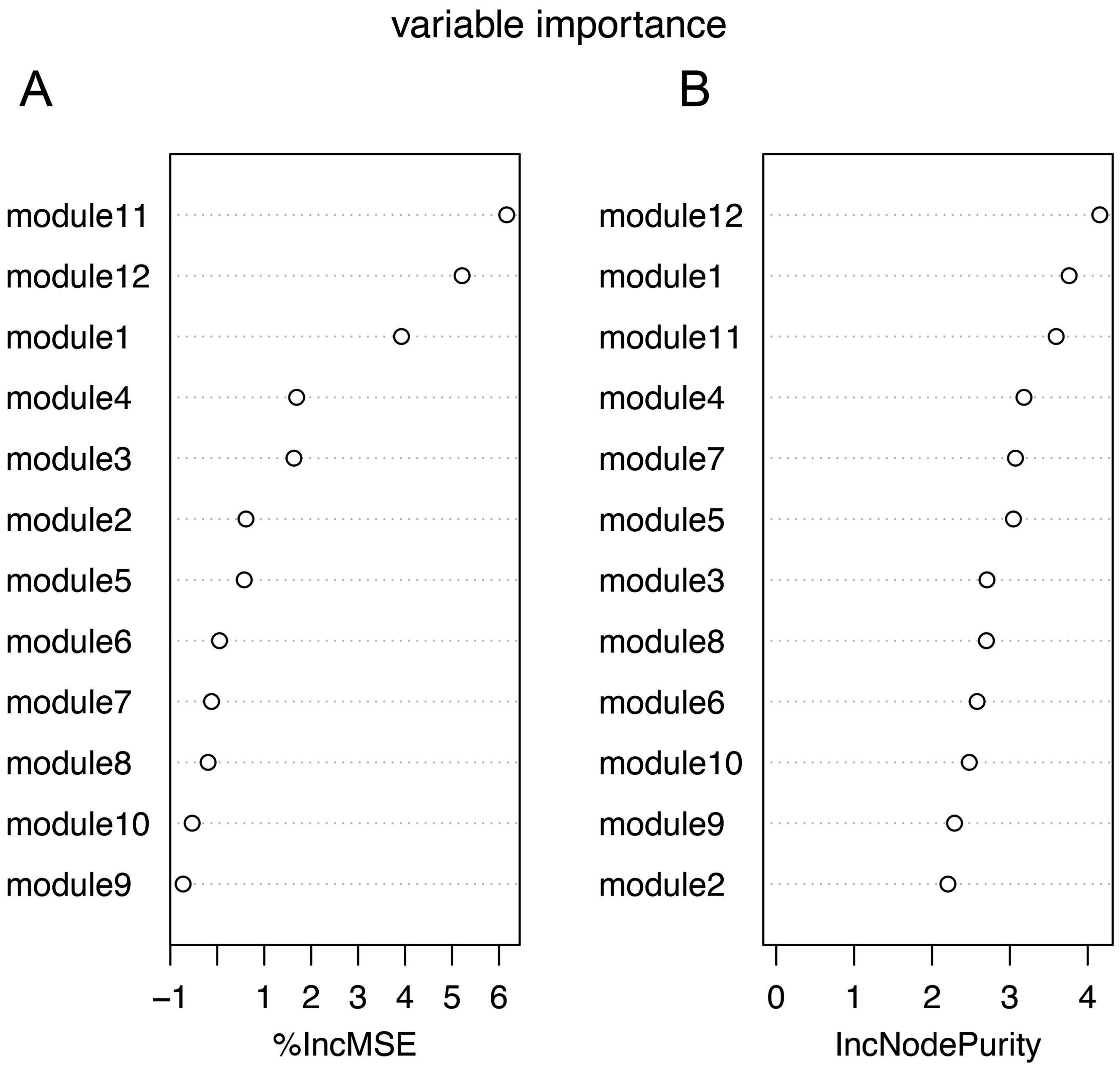
variable importance
A
module11
module12
module1
module4
module3
module2
module5
module6
module7
module8
module10
module9
−1
1
2
3
4
5
6
%IncMSE
B
module12
module1
module11
module4
module7
module5
module3
module8
module6
module10
module9
module2
0
1
2
3
4
IncNodePurity

Figure S4. A:%IncMSE is the most robust and informative measure. It is the increase in mse of predictions(estimated with out-of-bag-CV) as a result of variable j being permuted(values randomly shuffled). Figure B: IncNodePurity relates to the loss function which by best splits are chosen. The loss function is mse for regression and gini-impurity for classification. More useful variables achieve higher increases in node purities, that is to find a split which has a high inter node 'variance' and a small intra node 'variance'.

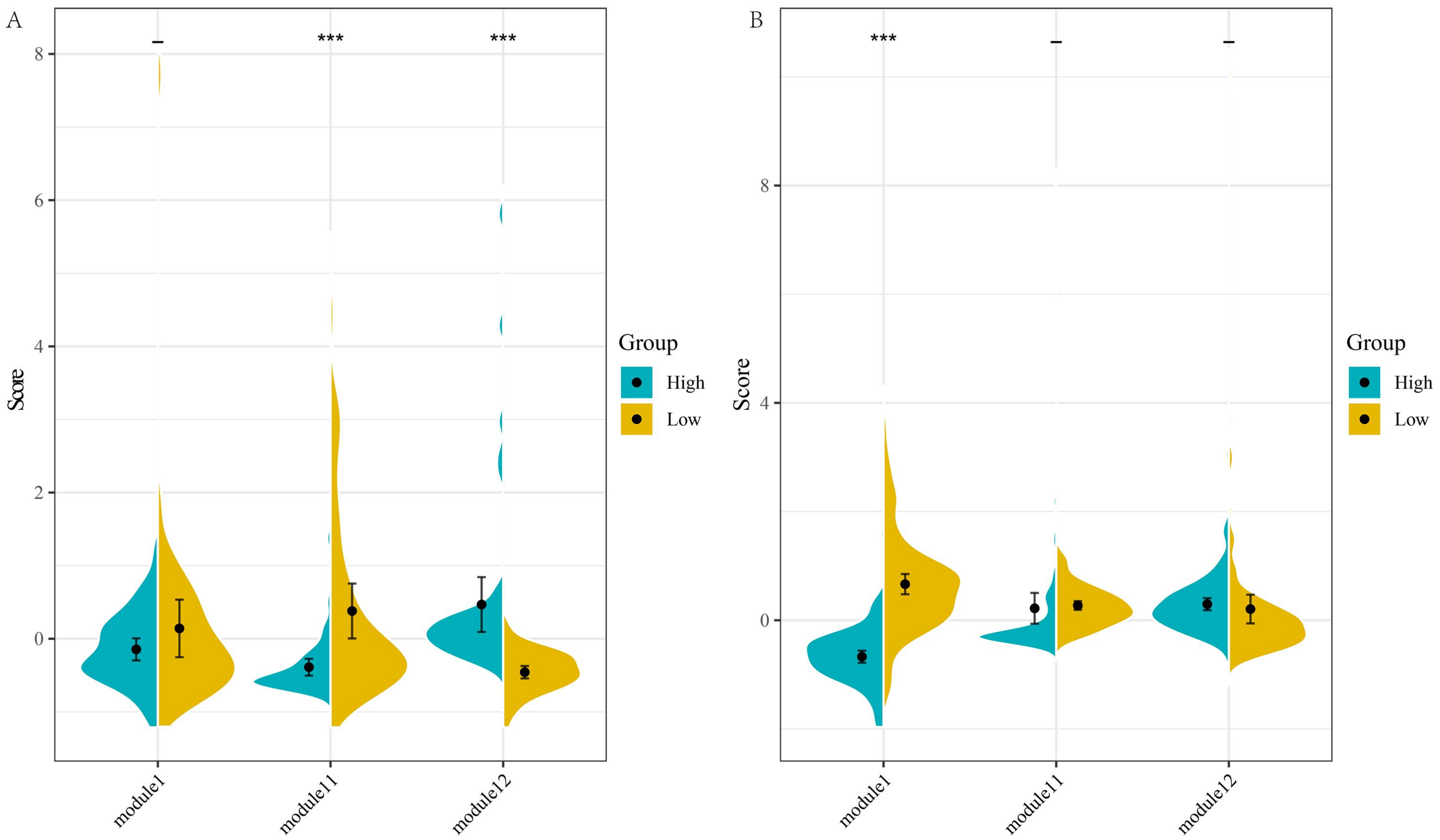

A
B
Score
Group
High
Low
***
module1
module11
module12
0
2
4
6
8

Figure S5. A. The Riskscore distribution of 3-PPI-MOD in ICGC-PAAD-AU.B.TheRiskscore distribution of 3-PPI-MOD in ICGC-PAAD-CA.

Table S1. Information of 3 datasets of PAAD

Database	usage	number of HCCs	Platform	organization
TCGA-PAAD	Traning	177	RNA-Seq	NIH
ICGC-PAAD-AU	Validation	212	RNA-Seq	ICGC
ICGC-PAAD-CA	Validation	184	RNA-Seq	ICGC
total		485		

Table S2. DEGs between tomor and normal (N vs T)

Genes	PPEE	PPDE	PostFC	RealFC	C1Mean	C2Mean
CD5L	0	1	35.79294546	1343.577417	5359.444963	3.978943915
FAM9C	0	1	2.147160355	63.8687062	89.19407471	1.386678906
GPA33	0	1	0.178593211	0.228190725	90.80653578	397.9752194
HOXA13	0	1	0.068598277	0.076299406	22.94872629	300.8930816
LMAN1L	0	1	8.600907641	311.2961135	121.4219157	0.380084908
LRTM1	0	1	1.297094848	24.88625508	27.29719531	1.087280214
NKX2-5	0	1	1.418094201	27.44640937	111.31186	4.045971712
NR5A1	0	1	35.34327679	1326.24527	1647.354031	1.232126225
OR5AK2	0	1	5.012107487	172.7716044	20.0620017	0.106176508
RASGRP2	0	1	1.610714426	5.639892396	3691.662408	654.5543337
SLC26A3	0	1	0.000832272	0.004301775	2.156782446	503.6849466
SLC9A4	0	1	0.017270174	0.018525937	4.321220804	233.7822624
SPIC	0	1	2.725486871	87.02122363	128.7588858	1.469741153
STAB2	0	1	1.359646031	27.31646505	1647.840403	60.31443801
HIST2H3C	1.11E-16	1	1.30517186	25.95212343	34.77732869	1.33044248
KRT72	1.44E-15	1	1.23650222	17.5268714	130.7867161	7.452639119
FCRL2	1.55E-15	1	1.195069221	17.67182301	2058.770913	116.4907658
FCRL1	2.33E-15	1	1.186564364	17.73787028	2764.895628	155.8658512
FCRL3	7.11E-15	1	1.204808631	17.61974172	3502.532292	198.7751098
KLHL14	1.67E-14	1	1.203759622	16.46495229	743.6120362	45.15393507
TEX101	1.55E-13	1	1.167121575	16.20282252	73.44756384	4.523627628
CHRNA4	2.29E-12	1	1.169207374	17.49996112	57.13171198	3.25524794
PAX5	2.49E-12	1	1.177238737	14.70409878	5179.033777	352.2076948
TNFRSF13B	3.72E-12	1	1.194039088	14.49701463	895.5880488	61.768102
ITGAD	4.88E-12	1	1.48663524	14.82947061	379.5231922	25.58317201
KRT75	5.83E-12	1	1.833436474	10.05653681	96.93517594	9.630016015
CNR2	6.14E-12	1	1.187179578	14.36368884	1295.918581	90.21254625
DNASE1L3	9.16E-12	1	1.269188745	15.03490473	1296.277059	86.20850837
GPR182	1.68E-11	1	1.303062644	14.38459148	198.9798627	13.82354286
LIPF	7.65E-11	1	0.881923305	12.54377554	277.5603229	22.11813215
ATP4B	1.03E-10	1	1.199246839	10.91752237	15.93359959	1.450367934
PSG8	1.12E-10	1	2.711731122	14.17441127	7.954624395	0.551901601
KCNT1	1.52E-10	1	1.320017309	12.99742374	316.4650841	24.33906258
P2RX5	2.12E-10	1	1.216464713	12.87310769	2877.663414	223.5314698
CD19	2.98E-10	1	1.191391567	12.60238977	3752.021731	297.7138285
KRT73	3.52E-10	1	1.261283233	13.72529876	33.80035655	2.453360335
PSG9	1.54E-09	0.999999998	1.222426003	9.531694396	13.43248298	1.400293115
AMTN	2.31E-09	0.999999998	2.022431613	10.44395253	417.8385381	39.99865925
HBA1	5.32E-09	0.999999995	2.32476897	10.46689264	93.60105649	8.933538425
LEFTY2	6.91E-09	0.999999993	2.240291711	7.85437225	137.0123363	17.43535807
ADRA1A	9.81E-09	0.99999999	1.532993353	12.16554124	106.7434819	8.765070486
CRHBP	1.06E-08	0.9999	1.346000775	11.37525944	287.5018741	25.26519268

		99989				
TLR10	1.11E-08	0.999999989	1.320640748	11.60922095	1625.487576	140.0077999
TLX1NB	1.20E-08	0.999999988	1.252529799	14.86139159	16.42620129	1.095966504
FCRL5	1.26E-08	0.999999987	1.239879467	12.04218212	3007.662073	249.7513839
BLK	1.28E-08	0.999999987	1.197821449	11.29409286	3466.333958	306.9065447
FAM129C	2.38E-08	0.999999976	1.21289614	11.10151346	2806.629677	252.8059505
HBA2	2.64E-08	0.999999974	2.409640421	9.591077527	3229.257699	336.6849845
FCRLA	2.68E-08	0.999999973	1.220889832	10.94466247	2343.606055	214.1232417
HBG1	3.63E-08	0.999999964	2.651362282	38.10497041	3.835018003	0.090905944
CASP14	5.21E-08	0.999999948	0.001151881	0.001772875	0.581507296	333.6329593
FCAMR	6.09E-08	0.999999939	1.207344008	10.37541173	640.3304729	61.70711444
FCER2	7.41E-08	0.999999926	1.218104232	10.36551046	2380.942039	229.6894489
SIGLEC11	8.69E-08	0.999999913	1.946126417	9.690517918	434.8520224	44.86500318
HBM	1.23E-07	0.999999877	2.07641974	14.36850896	8.016010236	0.548583376
TMPRSS15	1.35E-07	0.999999865	0.001114545	0.003562138	1.073229325	304.0852655
ALPP	1.56E-07	0.999999844	0.001937956	0.003172046	0.804965782	256.9111929
CR1	1.62E-07	0.999999838	1.351529375	11.07382757	2790.698505	251.9993876
DEFA6	1.92E-07	0.999999808	0.000709603	0.002569831	0.290753648	117.022459
PARP15	1.99E-07	0.999999801	1.345186018	10.54660193	2990.338743	283.5267034
ENSG00000233024	2.72E-07	0.999999728	1.707843369	10.05065803	28.21043673	2.797819812
ALPPL2	2.98E-07	0.999999702	0.00360334	0.005216842	1.239716446	239.5441967
GDF7	4.16E-07	0.999999584	1.594252006	9.476300256	983.1047191	103.7345725
FABP2	4.69E-07	0.999999531	0.002488498	0.00346777	0.691856731	202.3842434
ALPI	5.38E-07	0.999999462	0.001702108	0.002413157	0.290753648	124.6208047
TDGF1	5.75E-07	0.999999425	1.365878061	9.253325932	289.9579847	31.32662284
DEFA4	6.77E-07	0.999999323	1.209793348	12.83347297	13.08391415	1.010293898
BFSP2	1.00E-06	0.999999	1.385475618	9.379772234	78.17885239	8.325900962
OR6S1	1.09E-06	0.999998912	1.44301221	17.27056198	5.770092746	0.324678903

SOX3	1.23E-06	0.999998774	3.272335369	8.63585059	6.704100779	0.767468381
TINAG	1.35E-06	0.999998646	0.039064747	0.038236097	3.610642413	94.68173702
ZIC2	1.38E-06	0.999998621	0.004788571	0.003128716	0.257106067	85.36241583
TNFRSF13C	1.55E-06	0.999998454	1.242350244	9.348214477	3809.61105	407.5139245
NPY5R	2.45E-06	0.99999755	2.46383357	8.172241856	82.34562451	10.06748253
CD160	2.48E-06	0.999997516	1.462169362	9.292572541	184.687064	19.86577317
TLX1	2.71E-06	0.999997285	1.325610495	8.9157896	297.3623474	33.34345054
NLRP4	3.37E-06	0.999996628	1.288195626	9.320185478	63.68051196	6.823609922
LRRC31	4.01E-06	0.999995992	0.022738135	0.025280017	5.387061098	213.4811998
SERPINA9	4.41E-06	0.99999559	1.258174923	8.612800692	159.3396461	18.49149002
KLRF1	4.46E-06	0.999995542	1.584304397	8.426683442	253.0461434	30.02033698
CHP2	4.78E-06	0.999995215	0.006495631	0.008638501	0.936796491	109.5919423
MEP1B	4.91E-06	0.999995091	0.002013473	0.006090298	2.259770143	372.6762167
KLK5	5.31E-06	0.999994692	0.004170934	0.010503665	2.326029182	222.3913442
SLC7A10	5.65E-06	0.99999435	1.315048605	8.085000375	111.1602564	13.74018568
SCN11A	6.38E-06	0.999993623	1.599616809	8.889459413	178.9908945	20.1263082
NCR1	7.74E-06	0.999992262	1.307478728	9.403768008	67.00324377	7.11621193
KIR3DX1	9.19E-06	0.999990809	1.39836394	9.510042265	19.67013015	2.05940512
FGFBP2	1.02E-05	0.999989816	1.510438241	8.799945431	177.3587041	20.14565954
PSAPL1	1.06E-05	0.99998941	0.01765045	0.021822675	5.681610483	260.8017752
PSG5	1.07E-05	0.999989309	2.990133605	6.494049125	22.13341625	3.399801162
SLC4A1	1.09E-05	0.999989145	2.638420007	7.999797908	29.76014326	3.711361915
CLECL1	1.10E-05	0.999988967	1.82611682	8.326395589	322.9697066	38.77985848
NKX6-2	1.12E-05	0.999988834	0.009353096	0.009370859	0.838613363	90.54875932
CXCR5	1.50E-05	0.99998499	1.331002357	8.681584223	202.5578035	23.32304594
TM4SF19	1.54E-05	0.999984555	1.376644268	7.199640146	155.5534686	21.59711722
SCNN1G	1.57E-05	0.999984274	2.621100695	6.608765092	1084.053424	164.0241893
KIR2DL1	1.88E-05	0.9999	1.281554835	9.812570615	16.16909523	1.638813126

		81181				
ATOH1	1.95E-05	0.999980518	0.004059375	0.008670505	1.383713463	160.7318951
KRT79	2.11E-05	0.999978854	1.45318016	4.724670112	145.2447263	30.73388747
C6orf15	2.20E-05	0.999978028	0.007346615	0.014588599	4.166066311	286.245469
BPIFA2	2.22E-05	0.999977798	0.001448112	0.006751427	1.126607396	168.3406981
DEFA3	2.64E-05	0.999973569	1.317004946	10.01198659	14.77229835	1.466460063
APOA2	2.79E-05	0.999972066	0.007834398	0.009021729	1.080793507	120.8973622
ABCA12	3.07E-05	0.999969278	0.063608166	0.06436089	12.46974757	193.8926574
FABP6	3.09E-05	0.999969143	0.045283596	0.048049082	6.873468086	143.2490957
PSG3	3.19E-05	0.999968118	3.011881246	8.817389274	5.33974021	0.5967261
DYNAP	3.27E-05	0.999967276	1.28134064	19.05172821	3.779797421	0.188921451
SLCO1B3	3.35E-05	0.999966491	0.022679909	0.023869359	2.483053476	104.4357648
LYPD2	3.44E-05	0.999965612	0.014160182	0.021196284	6.31721051	298.4956434
UGT1A1	3.73E-05	0.999962701	0.019145704	0.021302675	1.285530335	60.80538187
LILRA4	3.78E-05	0.999962187	1.930451071	7.841521341	415.3148774	52.95483416
C20orf85	3.80E-05	0.99996197	0.001753232	0.004847022	0.355289195	75.35362822
C10orf90	4.38E-05	0.999956226	1.995893608	5.456694309	28.31054863	5.180055926
TNMD	5.68E-05	0.99994318	1.279102092	7.339539534	88.04893047	11.98788216
TDRD15	8.85E-05	0.999911503	1.277766556	13.46913544	7.525947262	0.549497475
IVL	0.000102289	0.999897711	0.020036555	0.022795845	1.453768239	64.20206196
PAEP	0.000102546	0.999897454	0.006982013	0.016940071	5.714732605	337.9302989
HOXA11	0.000129443	0.999870557	0.060933449	0.061477883	4.177223787	68.09943378
LRRC26	0.000132797	0.999867203	0.006890367	0.013724474	1.307011608	95.9508043
STAP1	0.000146938	0.999853062	1.364498135	7.531224996	720.3808974	95.64388072
C12orf42	0.000149897	0.999850103	1.339421556	7.611572851	89.99251243	11.81443027
KLRC4	0.000153163	0.999846837	1.849786776	7.777543119	25.20509472	3.232038564
HELT	0.000157575	0.999842425	1.400460465	25.29684219	2.61678283	0.093838369
KLK12	0.000160827	0.999839173	0.006203331	0.016262586	2.278289358	140.6988205

CACNG3	0.000161372	0.999838628	4.885427105	5.114838505	8.399846719	1.634205719
TBX21	0.000161946	0.999838054	1.672055126	7.551533332	421.9513883	55.8675774
MOGAT3	0.000180433	0.999819567	0.03646464	0.040147099	3.62096621	90.43155912
CTD-2207O23.3	0.000186097	0.999813903	1.425819257	7.978685893	42.4182548	5.307699602
BTLA	0.000190783	0.999809217	1.43725808	7.647401933	675.0933792	88.26878868
CALHM3	0.000192732	0.999807268	0.040413955	0.046309273	9.319588908	201.4526513
HOXA10	0.000195635	0.999804365	0.077858317	0.083829733	20.17645765	240.7930768
PRSS33	0.000197197	0.999802803	0.014428156	0.023671255	3.67675359	155.7381242
CYP3A4	0.000254171	0.999745829	0.013019564	0.028402038	4.274398083	150.8382607
DUSP27	0.000280438	0.999719562	0.014530816	0.025204628	9.169414137	364.1855825
IRX6	0.000286882	0.999713118	2.165658829	6.85021556	75.21287725	10.97109637
LHX5	0.00030225	0.99969775	0.004305187	0.024940788	1.798558484	72.50408662
KLRD1	0.000305923	0.999694077	1.567006858	7.529239657	1059.67297	140.7323616
KLRC4-KLRK1	0.000307158	0.999692842	1.70614175	7.47510763	46.57117326	6.22150536
NTS	0.000313349	0.999686651	0.005750511	0.03906042	13.62159638	348.9774537
CLEC17A	0.000315785	0.999684215	1.407344794	7.384892517	467.5002856	63.29630873
GPR128	0.000323687	0.999676313	0.042196943	0.049787549	7.525632549	151.3457657
MOGAT2	0.000326073	0.999673927	0.042907255	0.052296692	9.221461914	176.5109546
ZIC5	0.000351914	0.999648086	0.029658854	0.02058017	0.514212134	25.46171118
TRIM40	0.000353245	0.999646755	0.018961799	0.010924961	0.257106067	24.43915508
ADAP2	0.000364385	0.999635615	2.552646183	2.593072725	1308.346837	504.5484819
AC092159.1	0.000385448	0.999614552	1.261616377	13.84430976	4.070551069	0.284745721
SMYD1	0.000394719	0.999605281	0.008402623	0.022895659	1.564117675	68.74179459
FCRL4	0.000397961	0.999602039	1.372359748	6.759690561	104.6481357	15.47268146
SLC28A2	0.000407007	0.999592993	0.019143315	0.031649094	4.095919807	129.7226183
KCNJ10	0.000466001	0.999533999	1.455769075	7.061359367	222.3474998	31.47933347
FEZF1	0.000479337	0.999520663	0.052839986	0.056995356	4.661381633	81.95074183
CPN2	0.0005089	0.9994	1.496870575	7.020275032	52.82269308	7.51572981

	36	91064				
PRSS57	0.000512019	0.999487981	0.004201902	0.036094971	3.599393209	99.98712183
AMY1B	0.000539661	0.999460339	0.007018615	0.007078982	0.355289195	51.59193635
PADI6	0.000586197	0.999413803	2.764588039	13.39154322	3.452636578	0.248568898
TRAPPC3L	0.000588936	0.999411064	1.76643398	7.892867588	22.55722148	2.849191698
NMUR2	0.000627225	0.999372775	0.064633133	0.072392026	14.39615182	198.9919141
PAX7	0.000649299	0.999350701	0.011010654	0.028028066	1.163014591	41.84142844
AC234582.1	0.000678562	0.999321438	0.073978389	0.064100604	1.988544542	31.16824819
TRIM10	0.000688635	0.999311365	0.156638848	0.157596067	15.98755093	101.4998358
FCRL6	0.000725777	0.999274223	1.966060869	6.806783251	315.6719465	46.36755235
GGTLC1	0.000751462	0.999248538	2.567796596	5.461885543	35.29285089	6.453491519
SCGB1A1	0.00076467	0.99923533	0.009615731	0.011273292	0.290753648	26.66842354
LHX1	0.000775993	0.999224007	0.038298456	0.03854117	1.330335392	34.76671762
ADAD2	0.000809488	0.999190512	1.985246764	6.202088994	60.75201318	9.78702375
TPO	0.000812732	0.999187268	1.698732969	6.910447292	326.4197262	47.22713421
LCT	0.000820766	0.999179234	0.018009499	0.041153078	2.402731037	58.61820397
TTC24	0.000832745	0.999167255	1.678074593	6.843998439	88.27381381	12.88944973
SLC9A5	0.000872464	0.999127536	1.636131003	6.580470171	542.509221	82.43383865
CKMT1A	0.000894173	0.999105827	0.161024193	0.164751666	53.20553596	322.9945385
C11orf21	0.00089765	0.99910235	1.618520534	6.913956818	556.1320329	80.42759131
CCDC172	0.000969282	0.999030718	1.947590969	9.098349185	2.135328464	0.225793155
RETNLB	0.000990326	0.999009674	0.00560888	0.014084977	0.581507296	41.98561629
BEND4	0.001159991	0.998840009	1.361392533	6.644733941	222.4543815	33.46980272
ALAS2	0.001196604	0.998803396	3.111543653	5.497809366	71.54263063	13.00475294
PRAME	0.001228878	0.998771122	0.045267375	0.056877424	6.333914505	111.5266009
VPREB3	0.001376495	0.998623505	1.325300773	6.475520705	968.38868	149.5376154
GNGT1	0.001392076	0.998607924	0.044526169	0.022589028	0.290753648	13.30414712
HOXB13	0.001399664	0.998600336	0.038565638	0.043274317	5.290885778	122.4849617

CYP24A1	0.00140645	0.99859355	0.039328763	0.053258711	11.46479494	215.4438611
VSTM2A	0.001414339	0.998585661	0.007340977	0.032106046	6.72041313	209.6207094
GDPD2	0.001420866	0.998579134	1.408625774	6.544951724	774.276385	118.2928413
NOX5	0.001426108	0.998573892	1.331157683	6.394895799	390.8251308	61.10673169
SOSTDC1	0.001451974	0.998548026	0.072515122	0.08154407	13.16743174	161.588896
GIP	0.001456598	0.998543402	0.013757365	0.042080041	1.564117675	37.39770292
TUBAL3	0.001475195	0.998524805	0.051941648	0.04741595	1.095719429	23.30956693
ISX	0.001517949	0.998482051	0.012663728	0.040058419	1.163014591	29.27259852
CLCA4	0.001518668	0.998481332	0.015185879	0.032217313	1.821223742	56.82974146
UPK3BL	0.001595784	0.998404216	0.063234352	0.051966028	1.095544276	21.26436552
NPBWR1	0.001681596	0.998318404	0.034758834	0.037092533	1.205060034	32.74753714
PSG1	0.001690939	0.998309061	4.790828534	8.059784588	3.214910696	0.390123683
RETN	0.001772814	0.998227186	1.496775235	5.995982099	706.5133381	117.8227964
MUC21	0.001819575	0.998180425	0.027483757	0.041333925	3.566229001	86.51043132
SPRR2D	0.001832315	0.998167685	0.021851558	0.017889954	0.434750665	24.85035879
HS3ST5	0.001979928	0.998020072	0.087375259	0.086279688	3.284048368	38.16872348
CDHR1	0.00203584	0.99796416	1.688168852	6.131364384	239.3371975	39.02653127
TREML2	0.002096883	0.997903117	1.440846073	6.691164837	409.5781115	61.20327474
PTCRA	0.002106038	0.997893962	3.781334774	4.41279304	53.0878727	12.02271312
TMEM105	0.002175964	0.997824036	0.161763106	0.163949537	19.95299916	121.7530715
SAGE1	0.00226095	0.99773905	1.440909737	4.331887491	18.28945177	4.214359892
CD300LG	0.002321668	0.997678332	2.351527229	5.937772321	186.3711077	31.3790627
ZG16	0.002366799	0.997633201	0.027847664	0.049637292	4.191974556	84.64358184
KIF2B	0.002405608	0.997594392	1.325161679	13.92155804	1.744521887	0.116029133
PGA5	0.002485104	0.997514896	1.171050859	2.886431164	29.46130364	10.20029152
COL4A3	0.002528655	0.997471345	1.447041986	6.760252744	1183.011778	174.9866788
MPPED1	0.002555086	0.997444914	0.006259462	0.050166234	3.864897138	77.23114016
KLRK1	0.0025676	0.9974	1.80474937	6.188554526	139.1862515	22.48253052

	3	3237				
AKR1C4	0.002645117	0.997354883	0.043661734	0.060630328	5.491624425	90.73046896
PCDH11X	0.002667955	0.997332045	1.365603387	6.991561257	15.58758793	2.220916294
KEL	2.89E-03	0.997108087	2.119073203	7.045218996	355.5156707	50.45339524
EVX1	2.98E-03	0.997024791	0.019594486	0.031926797	2.778584402	87.33306674
LILRA1	2.99E-03	0.997014944	2.671222841	6.122600842	304.0421522	49.65061974
KIR3DL1	3.08E-03	0.996923875	1.894438304	6.789194787	13.51673888	1.982392221
KIR2DL3	3.10E-03	0.996904361	1.697043699	7.306142	13.20625474	1.798923881
WISP3	3.12E-03	0.996877875	0.079502843	0.090144557	15.27949256	169.600823
OR52N4	3.13E-03	0.996873223	1.508507528	6.277428017	44.13254724	7.021947977
PGLYRP4	3.28E-03	0.996723393	2.616764502	4.525114565	77.70852932	17.16493076
LEP	3.30E-03	0.996695025	1.264377761	6.292622456	796.3796382	126.5492595
ITLN2	3.35E-03	0.996648337	0.014707654	0.033962846	0.547859715	16.41558803
GHRH	3.39E-03	0.996613305	0.007011428	0.019212843	0.290753648	15.64378154
IRX1	3.41E-03	0.996593644	0.016501449	0.037092012	2.902675775	78.51568867
AQP6	3.56E-03	0.996442409	0.097520704	0.103205397	8.652590251	83.92543826
GP9	3.56E-03	0.996435544	1.20517141	6.16870398	14.24692874	2.301170837
ABCG8	3.74E-03	0.996255497	0.055034905	0.057838817	1.386297924	24.1311911
IDI2	3.83E-03	0.996170027	1.466424807	8.736404123	4.549322753	0.511876357
ASCL1	3.95E-03	0.996050424	0.007332112	0.032293911	4.670723889	144.9313753
NEK2	4.03E-03	0.995968699	0.254398969	0.259511641	108.1557514	416.7950071
TMEM211	4.07E-03	0.99593263	0.03664307	0.043242764	1.273188874	29.66407179
EOMES	4.43E-03	0.99557408	1.989383573	6.288491777	557.4194024	88.63278147
CREB3L3	4.51E-03	0.995485267	0.032587309	0.058802456	12.59140234	214.2906142
GJB5	4.54E-03	0.995462543	0.079561625	0.083510887	33.23851549	398.1239018
PPP1R14D	4.58E-03	0.995423054	0.055821255	0.058906535	21.25681742	361.0164538
HOXC11	4.63E-03	0.995371543	0.124634234	0.132912084	16.48843587	124.1204426
APOBEC1	4.71E-03	0.995288334	0.057205692	0.071589735	19.07444975	266.5708143

GPR18	4.81E-03	0.995190808	1.598208994	6.279408096	319.6612567	50.89786454
TMEM82	4.93E-03	0.995071631	0.070674436	0.072952096	2.011034647	27.69358561
ALG1L	5.08E-03	0.994922026	0.108472828	0.110429746	13.46557442	122.018483
APOA1	5.17E-03	0.99482871	0.035323214	0.064831756	15.59029141	240.6173153
BANK1	5.34E-03	0.994658643	1.422450682	6.539325552	4088.408427	625.1949075
GATA5	5.37E-03	0.99462996	0.0277824	0.049648471	4.470589187	90.23626719
HAPLN1	5.39E-03	0.994605553	0.060791227	0.075580748	9.772333329	129.418904
CFP	5.67E-03	0.994330338	1.697826338	6.29959063	1218.614687	193.4350599
MTTP	5.67E-03	0.994326725	0.017513499	0.072063974	9.152499395	127.1339651
AIF1L	5.73E-03	0.994273695	1.680361207	6.31214264	3736.050148	591.87462
OTX1	5.76E-03	0.994238902	0.224152369	0.226101016	23.20910683	102.6835097
GLYAT	6.15E-03	0.99384859	1.326616616	5.826462093	27.06343219	4.636633198
ATP4A	6.16E-03	0.993843411	1.390017616	2.761069567	31.47707997	11.393943
SLC10A2	6.16E-03	0.993839901	0.009591405	0.043691632	2.972072025	68.24270417
CD79B	6.17E-03	0.993828962	1.447363593	6.222225706	3892.474497	625.5675154
CCDC108	6.22E-03	0.993779662	0.081836769	0.089882182	8.095351134	90.1675073
HOXD10	6.23E-03	0.993774363	0.040256119	0.036052548	0.691856731	19.45760411
HRG	6.46E-03	0.993535605	0.039752908	0.034064728	0.581507296	17.35421582
CASP16	6.53E-03	0.993473039	1.546543764	6.157029792	90.01735571	14.6118808
SH3TC2	6.60E-03	0.993404788	0.160702949	0.164787078	30.77515124	186.8077502
ELANE	6.74E-03	0.993261217	0.008345691	0.067206796	10.08855394	150.250904
TNNI3	7.01E-03	0.992987944	0.096407004	0.103419799	5.912080402	57.2525403
DEFB132	7.14E-03	0.992862881	1.180115573	8.027174285	5.91343954	0.727923375
SHISA8	7.31E-03	0.992691186	1.368414421	5.250673891	57.45677174	10.9346469
PF4V1	7.54E-03	0.992456169	3.18068668	5.105991886	30.53603063	5.972389183
SFTPC	7.64E-03	0.992355837	0.007645899	0.031800391	0.646042843	20.6200246
DCSTAMP	7.65E-03	0.992354039	2.399176136	4.939087541	348.1564853	70.48206607
TUBB4A	7.86E-03	0.9921	3.266786327	3.665802772	276.9989507	75.55569951

		41794				
TMEM151A	7.98E-03	0.992017204	0.083117434	0.09519398	20.09738627	211.2153977
EN2	8.07E-03	0.99192963	0.080867141	0.086629302	3.537736295	40.94307502
MSLNL	8.18E-03	0.991824685	0.056534568	0.072345791	6.99782634	96.85570866
ENTPD8	8.20E-03	0.991803279	0.094003882	0.106260588	62.4227578	587.5338744
GTSF1L	8.41E-03	0.991588374	1.646296767	6.461069924	13.7196332	2.114978271
NPY1R	8.63E-03	0.991372941	2.00571431	5.430920909	1303.385878	239.9853709
CALML3	8.76E-03	0.991239372	0.01641243	0.054731385	4.214548086	77.17693905
CDH12	8.88E-03	0.991124553	0.011625396	0.060748945	4.251732825	70.14319889
CDH16	8.95E-03	0.991052624	0.037639506	0.055424929	3.114143106	56.3571108
TBX4	9.02E-03	0.990984428	0.018069728	0.040601396	0.982435226	24.4333772
IGFL1	9.07E-03	0.990933856	0.042550224	0.063898342	9.61386722	150.6021583
NKX6-3	9.08E-03	0.99091981	0.010596771	0.073258583	23.75094889	324.3335502
KRT4	9.34E-03	0.990656184	0.044068995	0.067779385	23.92062542	353.0564264
PLA2G3	9.42E-03	0.990576957	0.068833482	0.069937053	1.719272168	24.71612279
GBX1	9.50E-03	0.990503937	0.00816772	0.041456937	0.581507296	14.25799316
KISS1	9.62E-03	0.990378105	0.021189948	0.064866173	6.627611223	102.3177765
CKMT1B	9.72E-03	0.990276684	0.16572571	0.171038459	68.43491653	400.1626678
OR2L13	9.80E-03	0.990202964	1.454536956	5.841208811	16.89183992	2.883551741
SLC6A19	1.03E-02	0.989701366	0.021301143	0.059953191	24.21786843	404.1030739
SEMA3D	1.05E-02	0.989533616	1.591386648	6.241620807	966.3188018	154.8101712
SLC36A3	1.05E-02	0.989459742	1.353761837	8.888822664	4.652058365	0.514485474
AHSG	1.06E-02	0.989435611	0.092491037	0.08758991	2.011034647	23.06382938
KLF1	1.07E-02	0.989328478	4.075884668	4.437901543	10.44314252	2.345424612
AJAP1	1.08E-02	0.989242422	2.878488887	4.056009005	569.3717552	140.3698055
DSC3	1.09E-02	0.989064948	0.090535602	0.104173945	28.64057303	275.0162845
SLC13A2	1.10E-02	0.988980913	0.051580098	0.0792287	19.23964636	242.9530471
CX3CR1	1.15E-02	0.988516998	3.606117961	4.445245064	751.8984598	169.1389331

FTCD	1.16E-02	0.98844977	0.015393838	0.060415779	18.80658698	311.4415351
COL19A1	1.17E-02	0.988283462	1.410020731	5.872016417	125.8289782	21.42028378
UGT2B4	1.18E-02	0.98819296	0.021132094	0.057104295	2.60470825	45.77829416
RTBDN	1.20E-02	0.987983772	0.014127596	0.044655663	8.870114766	198.847527
PNCK	1.24E-02	0.987604571	0.043305567	0.068297517	13.98578916	204.9138362
PIP	1.26E-02	0.987365463	0.016372994	0.040951957	3.152567965	77.21629697
ACSM5	1.27E-02	0.987341359	3.061820322	3.159405388	147.2595787	46.60306816
TCL1A	1.29E-02	0.987067334	1.316516867	5.788464447	1993.201784	344.3320621
PATL2	1.33E-02	0.986680753	1.667079473	5.746450336	592.1119156	103.0313353
CNTD2	1.37E-02	0.986291716	0.056044566	0.077372975	5.421275477	70.18602748
KRT82	1.37E-02	0.986253096	2.060721307	10.60223873	2.481183675	0.224967702
HTRA4	1.40E-02	0.986011939	3.169362031	3.486163148	169.7271576	48.67881645
CEACAM20	1.43E-02	0.985738641	0.042326102	0.076927578	1.677051572	21.92038727
RHCG	1.45E-02	0.985492958	0.057083604	0.080162777	9.111581803	113.7782465
PTGDR	1.47E-02	0.985305286	1.896530421	5.57316279	227.2374454	40.7653109
HRASLS2	1.48E-02	0.985224633	0.067247419	0.071009962	17.48858392	246.4143526
OVOL1	1.49E-02	0.985149646	0.158983767	0.165028638	75.06593455	454.9167042
EMR1	1.52E-02	0.984780911	2.872856167	5.139833608	255.0085806	49.60611602
SIGLEC9	1.53E-02	0.984691815	2.661044369	2.729697385	519.7323488	190.3929185
KRTAP4-1	1.56E-02	0.984442923	0.082890727	0.064035162	0.771318201	12.19139334
UGT1A8	1.58E-02	0.984173159	0.094199678	0.054004863	0.434750665	8.22538171
MLN	1.59E-02	0.984123247	0.01748869	0.099361861	2.01278543	20.3477652
FXYD4	1.70E-02	0.982979575	0.060230065	0.04931679	0.827280734	16.96760007
UNC45B	1.70E-02	0.982976282	1.64907473	5.747322655	33.5190096	5.823848491
ZNF560	1.71E-02	0.982890203	2.0730142	6.082491681	10.34076371	1.691730845
MAGEA4	1.74E-02	0.982624268	0.009672066	0.039751708	0.581507296	14.87004722
H3F3C	1.75E-02	0.982476437	2.031719825	5.296449124	72.01833741	13.5893636
HOXC13	1.78E-02	0.9821	0.036609644	0.039923249	0.547859715	13.96330454

		65176				
SLC5A10	1.80E-02	0.98199273	1.78572971	5.157915959	102.7173484	19.90644478
OR2A42	1.84E-02	0.981639848	1.900057341	9.756273669	3.153309916	0.314233414
KMO	1.85E-02	0.981508929	3.211875977	4.980277955	480.367553	96.4459724
TBX15	1.89E-02	0.981132488	0.12471882	0.13912498	37.29114973	268.102526
ACTL8	1.90E-02	0.98103387	0.025833991	0.02558594	0.468398245	18.68770033
NCR3	1.91E-02	0.980909513	1.670768424	5.511076421	229.2658086	41.59272714
HK3	1.93E-02	0.980737491	2.775940963	5.170787786	2191.761244	423.8656908
FAM83C	1.93E-02	0.98071107	0.058418556	0.029123766	0.257106067	9.161412335
ZC3H12D	1.98E-02	0.98015229	1.803997596	5.540816646	819.8949596	147.9654722
CHRM2	2.00E-02	0.98003664	0.040607168	0.077082362	4.120252422	53.57232852
PIWIL1	2.05E-02	0.979508694	0.097322876	0.114722545	8.251276421	72.00092356
HIST1H2BC	2.06E-02	0.979432532	0.1561342	0.160800293	27.4000502	170.4502003
SPERT	2.12E-02	0.978829317	0.097370864	0.088088828	1.352650343	15.45904836
DKK4	2.14E-02	0.9785776	0.07125826	0.087436878	7.395336131	84.68351034
UPK2	2.19E-02	0.978100748	0.060448655	0.089037387	7.51556733	84.51143073
TEKT1	2.19E-02	0.978060255	0.030060635	0.060264077	3.413469769	56.79780212
SIGLEC14	2.20E-02	0.978033876	2.187124228	5.241477279	1014.895996	193.6197616
ZPBP2	2.20E-02	0.978027529	1.374303836	5.647803071	10.06326862	1.773572921
ELF5	2.23E-02	0.977655144	0.090673562	0.107509979	10.94043417	101.8450485
TMEM213	2.25E-02	0.977474354	0.040071022	0.065197964	3.208557789	49.35592518
AMHR2	2.29E-02	0.977050452	2.648292749	3.596483515	118.6355401	32.97931848
SCN4A	2.31E-02	0.976943438	2.819028905	4.89746052	135.3560673	27.63005278
CALCA	2.35E-02	0.976545643	0.01037215	0.052554984	4.429377423	84.46110238
CAMP	2.41E-02	0.975881656	2.439031183	4.996295829	69.29430438	13.86113709
SCIMP	2.46E-02	0.975391987	2.342583028	5.578010671	1647.153038	295.2857847
AIM2	2.48E-02	0.975248276	1.513139958	5.397084761	768.4867274	142.3810799
F2	2.53E-02	0.974699758	0.086140113	0.086068213	1.854871323	21.65736418

IL13	2.54E-02	0.974578581	1.728892417	4.806097619	15.22061836	3.159019767
LCN1	2.56E-02	0.9744412	0.013129143	0.08220493	3.004627349	36.66209933
CTD-3214H19.16	2.60E-02	0.973976036	1.370119512	6.376617549	4.627909205	0.717330622
SPATA21	2.60E-02	0.973960631	3.113360764	4.177507287	23.0623271	5.512989072
MMP3	2.62E-02	0.973826744	0.019913507	0.078044865	32.2683906	413.5776304
BTNL2	2.65E-02	0.973511696	1.599553067	6.113639357	9.044251048	1.470992011
SIGLEC7	2.65E-02	0.973457594	3.10363413	3.221371269	341.1211875	105.8862656
ZNF716	2.66E-02	0.973389222	2.03954934	8.958424914	2.481183675	0.268082777
OR52N2	2.67E-02	0.973269704	2.488580016	3.554519627	17.52813096	4.92403689
CETP	2.69E-02	0.973149161	1.915082209	5.218071347	515.732572	98.82777697
CCR6	2.71E-02	0.972869408	1.752655966	5.518858039	97.75336828	17.70441983
CYP4F2	2.79E-02	0.97214935	0.078986209	0.106634878	4.783805649	44.94532541
PRR33	2.80E-02	0.971968286	2.075415616	5.207605653	231.134408	44.37592771
TLCD1	2.88E-02	0.971191074	0.306916197	0.3118511	147.9068472	474.3088241
ZNF488	2.91E-02	0.970904235	0.110721673	0.115898805	21.8719723	188.7923985
TPSG1	2.95E-02	0.970489733	0.028901401	0.066878477	13.52787581	202.4150029
OR6K2	2.99E-02	0.970062191	2.446747913	56.17529611	0.872260943	0.005705497
PLA2G10	3.00E-02	0.97004501	0.1217576	0.127330303	33.65564924	264.3862078
ARHGAP40	3.05E-02	0.969546723	0.0682616	0.091694751	13.03873825	142.2962723
PZP	3.10E-02	0.969024064	2.060308698	5.03101214	54.04469515	10.73429829
FOXE1	3.12E-02	0.968784955	0.082134674	0.080945558	1.337899574	16.64192762
KRTAP10-12	3.16E-02	0.968374543	1.40782256	13.33564585	1.453768239	0.099763581
LILRB5	3.18E-02	0.968221337	1.998639532	5.865241468	1216.343368	207.3733404
GAPT	3.20E-02	0.968007662	2.641890649	5.127753551	856.1976548	166.9651961
UBE2T	3.20E-02	0.968002208	0.36904064	0.374153943	162.6821949	434.8168889
SOX1	3.24E-02	0.967574316	0.007945633	0.015187282	0.177644598	12.34537735
EMX1	3.33E-02	0.966735308	0.040086926	0.088226428	3.922702181	44.56510379
SSTR5	3.35E-02	0.9665	0.122595041	0.126592517	5.083993281	40.22929226

		09708				
UGT2B11	3.38E-02	0.966241689	0.016979987	0.053539391	5.419929582	101.4093385
CCL15	3.39E-02	0.966084379	0.164603897	0.177288884	20.61230887	116.3103715
CAPSL	3.40E-02	0.966038912	0.04946291	0.071171907	3.443348904	48.5112363
CALML5	3.40E-02	0.96603426	0.053895883	0.077034448	1.965220759	25.63074645
SERPINA11	3.46E-02	0.965382216	0.043224978	0.070930218	2.099811082	29.73488372
SLC12A3	3.46E-02	0.965382069	1.616336614	5.047330306	71.70364435	14.19823287
HEATR9	3.47E-02	0.965342907	2.157757748	5.071754654	29.79871187	5.867396266
S100A7	3.58E-02	0.964191426	0.030463526	0.066726062	1.307011608	19.72758943
OR5B21	3.58E-02	0.964162917	1.702470326	15.55311834	1.631412837	0.095535932
CNNM1	3.59E-02	0.964147463	0.182667024	0.188746067	78.54094973	416.1626447
SPACA4	3.60E-02	0.96400193	0.115357798	0.116036217	7.11832442	61.42189267
SALL3	3.70E-02	0.963032702	0.010632345	0.023445633	0.177644598	7.993392218
FEV	3.78E-02	0.962162856	0.029557833	0.078227606	21.44684821	274.2774189
OR6K3	3.79E-02	0.962123498	2.163426741	9.212144252	2.381249764	0.249575805
TXK	3.79E-02	0.962076911	1.723253817	5.225563352	448.4190043	85.80448049
CHST5	3.79E-02	0.962060387	0.058983978	0.093410552	3.630939702	38.96782014
CRTAC1	3.80E-02	0.962023033	2.128762188	3.626285101	1129.892067	311.5766612
TMEM217	3.89E-02	0.961142047	2.712063477	3.946776338	209.2503704	53.01057997
TBATA	3.89E-02	0.961064342	2.296437883	5.062449083	8.603981155	1.691544255
INSL4	4.01E-02	0.959912796	0.017339668	0.050636515	0.936796491	18.68789983
NMU	4.03E-02	0.959738299	0.079784101	0.085328499	38.66667763	453.2579966
CCM2L	4.04E-02	0.959634268	2.059645037	5.064971252	837.1965819	165.2834519
SPATC1	4.06E-02	0.959352039	2.892235394	4.557594344	86.48755077	18.9687735
GPSM2	4.15E-02	0.958511281	0.457181394	0.461661676	244.3746474	529.3487491
AC108938.5	4.20E-02	0.958034714	2.077190082	4.541342837	136.5878748	30.06874095
FRMD4A	4.27E-02	0.957297222	2.070704993	2.099268165	1480.352119	705.1700929
DRC1	4.28E-02	0.957165093	0.074535948	0.099095184	7.474382857	75.51721091

NUP62CL	4.33E-02	0.956673382	0.314390614	0.318400909	51.21617937	160.8757825
MIOX	4.36E-02	0.956404886	0.160676055	0.173342199	11.74486618	67.80306731
MUCL1	4.38E-02	0.956204853	0.023214278	0.072991859	1.900685211	26.16668924
OR2A1	4.41E-02	0.955930657	2.932835373	6.348726156	5.174493207	0.806619441
SERTM1	4.49E-02	0.955114371	0.049012191	0.094830136	8.898566133	93.93235336
SP140	4.55E-02	0.95445289	1.732446994	5.408533558	1741.374321	321.9597728
SLC9A2	4.60E-02	0.954022989	0.08690557	0.09307815	45.34146607	487.2307311
RFX4	4.62E-02	0.953786147	2.709933258	3.596470708	13.11689516	3.639938015
KRT12	4.66E-02	0.953391731	1.439661109	4.406129544	53.13588343	12.05180683
SIM2	4.73E-02	0.952667973	0.152604322	0.159163511	74.52944957	468.3099624
CHI3L2	4.74E-02	0.952617524	2.208649288	4.922815307	953.0677785	193.594212
CD300LB	4.85E-02	0.951545423	2.225449922	4.597561895	354.3161496	77.05827177
MEP1A	4.85E-02	0.95147686	0.04156177	0.093538883	25.20906323	269.6004815
TMEM40	4.85E-02	0.951472095	0.089133428	0.118287825	12.6194951	106.759189
NKG7	4.90E-02	0.95095832	2.076466165	5.099118102	1445.469157	283.4663048

Table S3.Exosomes related genes

Gene	Species	Gene Name
LYPD2	Homo sapiens	LY6/PLAUR domain containing 2(LYPD2)
KLRC4	Homo sapiens	killer cell lectin like receptor C4(KLRC4)
CYP3A4	Homo sapiens	cytochrome P450 family 3 subfamily A member 4(CYP3A4)
CYP24A1	Homo sapiens	cytochrome P450 family 24 subfamily A member 1(CYP24A1)
ALPPL2	Homo sapiens	alkaline phosphatase, placental like 2(ALPPL2)
SLC9A5	Homo sapiens	solute carrier family 9 member A5(SLC9A5)
SERTM1	Homo sapiens	serine rich and transmembrane domain containing 1(SERTM1)
GDF7	Homo sapiens	growth differentiation factor 7(GDF7)
CRHBP	Homo sapiens	corticotropin releasing hormone binding protein(CRHBP)
SLC9A2	Homo sapiens	solute carrier family 9 member A2(SLC9A2)
DNASE1L3	Homo sapiens	deoxyribonuclease 1 like 3(DNASE1L3)
HOXD10	Homo sapiens	homeobox D10(HOXD10)
TMEM151A	Homo sapiens	transmembrane protein 151A(TMEM151A)
CFP	Homo sapiens	complement factor properdin(CFP)
RTBDN	Homo sapiens	retbindin(RTBDN)
LILRA1	Homo sapiens	leukocyte immunoglobulin like receptor A1(LILRA1)
SOSTDC1	Homo sapiens	sclerostin domain containing 1(SOSTDC1)
NKX6-2	Homo sapiens	NK6 homeobox 2(NKX6-2)
LILRA4	Homo sapiens	leukocyte immunoglobulin like receptor A4(LILRA4)
NKX6-3	Homo sapiens	NK6 homeobox 3(NKX6-3)
AIF1L	Homo sapiens	allograft inflammatory factor 1 like(AIF1L)
CHRNA4	Homo sapiens	cholinergic receptor nicotinic alpha 4 subunit(CHRNA4)
HTRA4	Homo sapiens	HtrA serine peptidase 4(HTRA4)
CREB3L3	Homo sapiens	cAMP responsive element binding protein 3 like 3(CREB3L3)
KLRD1	Homo sapiens	killer cell lectin like receptor D1(KLRD1)
PRAME	Homo sapiens	preferentially expressed antigen in melanoma(PRAME)
EMX1	Homo sapiens	empty spiracles homeobox 1(EMX1)
CRTAC1	Homo sapiens	cartilage acidic protein 1(CRTAC1)
CDHR1	Homo sapiens	cadherin related family member 1(CDHR1)
EOMES	Homo sapiens	eomesodermin(EOMES)
SPATC1	Homo sapiens	spermatogenesis and centriole associated 1(SPATC1)
RETN	Homo sapiens	resistin(RETN)
SSTR5	Homo sapiens	somatostatin receptor 5(SSTR5)
CCR6	Homo sapiens	C-C motif chemokine receptor 6(CCR6)
LILRB5	Homo sapiens	leukocyte immunoglobulin like receptor B5(LILRB5)
PARP15	Homo sapiens	poly(ADP-ribose) polymerase family member

		15(PARP15)
CX3CR1	Homo sapiens	C-X3-C motif chemokine receptor 1(CX3CR1)
F2	Homo sapiens	coagulation factor II, thrombin(F2)
HOXA13	Homo sapiens	homeobox A13(HOXA13)
SERPINA11	Homo sapiens	serpin family A member 11(SERPINA11)
NEK2	Homo sapiens	NIMA related kinase 2(NEK2)
HOXA11	Homo sapiens	homeobox A11(HOXA11)
KEL	Homo sapiens	Kell blood group, metallo-endopeptidase(KEL)
FCER2	Homo sapiens	Fc fragment of IgE receptor II(FCER2)
HOXB13	Homo sapiens	homeobox B13(HOXB13)
PF4V1	Homo sapiens	platelet factor 4 variant 1(PF4V1)
AHSG	Homo sapiens	alpha 2-HS glycoprotein(AHSG)
OR52N4	Homo sapiens	olfactory receptor family 52 subfamily N member 4 (gene/pseudogene)(OR52N4)
DYNAP	Homo sapiens	dynactin associated protein(DYNAP)
LHX1	Homo sapiens	LIM homeobox 1(LHX1)
PIP	Homo sapiens	prolactin induced protein(PIP)
HOXA10	Homo sapiens	homeobox A10(HOXA10)
KLRF1	Homo sapiens	killer cell lectin like receptor F1(KLRF1)
SLC28A2	Homo sapiens	solute carrier family 28 member 2(SLC28A2)
NKX2-5	Homo sapiens	NK2 homeobox 5(NKX2-5)
PTCRA	Homo sapiens	pre T-cell antigen receptor alpha(PTCRA)
TUBB4A	Homo sapiens	tubulin beta 4A class IVa(TUBB4A)
UNC45B	Homo sapiens	unc-45 myosin chaperone B(UNC45B)
CHI3L2	Homo sapiens	chitinase 3 like 2(CHI3L2)
CD5L	Homo sapiens	CD5 molecule like(CD5L)
HBA2	Homo sapiens	hemoglobin subunit alpha 2(HBA2)
EN2	Homo sapiens	engrailed homeobox 2(EN2)
HBA1	Homo sapiens	hemoglobin subunit alpha 1(HBA1)
TNNI3	Homo sapiens	troponin I3, cardiac type(TNNI3)
TRAPPC3L	Homo sapiens	trafficking protein particle complex 3 like(TRAPPC3L)
DCSTAMP	Homo sapiens	dendrocyte expressed seven transmembrane protein(DCSTAMP)
ABCG8	Homo sapiens	ATP binding cassette subfamily G member 8(ABCG8)
BTLA	Homo sapiens	B and T lymphocyte associated(BTLA)
GNGT1	Homo sapiens	G protein subunit gamma transducin 1(GNGT1)
CKMT1A	Homo sapiens	creatine kinase, mitochondrial 1A(CKMT1A)
ADAP2	Homo sapiens	ArfGAP with dual PH domains 2(ADAP2)
DUSP27	Homo sapiens	dual specificity phosphatase 27 (putative)(DUSP27)
KLRC4-KLRK1	Homo sapiens	KLRC4-KLRK1 readthrough(KLRC4-KLRK1)
IGFL1	Homo sapiens	IGF like family member 1(IGFL1)
H3F3C	Homo sapiens	H3 histone family member 3C(H3F3C)
IDI2	Homo sapiens	isopentenyl-diphosphate delta isomerase 2(IDI2)
TREML2	Homo sapiens	triggering receptor expressed on myeloid cells like 2(TREML2)
KLF1	Homo sapiens	Kruppel like factor 1(KLF1)

ZNF488	Homo sapiens	zinc finger protein 488(ZNF488)
PZP	Homo sapiens	PZP, alpha-2-macroglobulin like(PZP)
CKMT1B	Homo sapiens	creatine kinase, mitochondrial 1B(CKMT1B)
HELT	Homo sapiens	heltbHLH transcription factor(HELT)
ELF5	Homo sapiens	E74 like ETS transcription factor 5(ELF5)
TBX21	Homo sapiens	T-box 21(TBX21)
TTC24	Homo sapiens	tetratricopeptide repeat domain 24(TTC24)
FCRL2	Homo sapiens	Fc receptor like 2(FCRL2)
FCRL1	Homo sapiens	Fc receptor like 1(FCRL1)
KCNJ10	Homo sapiens	potassium voltage-gated channel subfamily J member 10(KCNJ10)
FCRL6	Homo sapiens	Fc receptor like 6(FCRL6)
SCIMP	Homo sapiens	SLP adaptor and CSK interacting membrane protein(SCIMP)
FCRL5	Homo sapiens	Fc receptor like 5(FCRL5)
FCRL4	Homo sapiens	Fc receptor like 4(FCRL4)
FCRL3	Homo sapiens	Fc receptor like 3(FCRL3)
KISS1	Homo sapiens	KiSS-1 metastasis-suppressor(KISS1)
SERPINA9	Homo sapiens	serpin family A member 9(SERPINA9)
SEMA3D	Homo sapiens	semaphorin 3D(SEMA3D)
FAM129C	Homo sapiens	family with sequence similarity 129 member C(FAM129C)
CETP	Homo sapiens	cholesteryl ester transfer protein(CETP)
SPIC	Homo sapiens	Spi-C transcription factor(SPIC)
PRSS33	Homo sapiens	protease, serine 33(PRSS33)
C12orf42	Homo sapiens	chromosome 12 open reading frame 42(C12orf42)
GAPT	Homo sapiens	GRB2 binding adaptor protein, transmembrane(GAPT)
TBX15	Homo sapiens	T-box 15(TBX15)
GIP	Homo sapiens	gastric inhibitory polypeptide(GIP)
ATP4A	Homo sapiens	ATPase H+/K+ transporting alpha subunit(ATP4A)
ATP4B	Homo sapiens	ATPase H+/K+ transporting beta subunit(ATP4B)
PCDH11X	Homo sapiens	protocadherin 11 X-linked(PCDH11X)
CAMP	Homo sapiens	cathelicidin antimicrobial peptide(CAMP)
ELANE	Homo sapiens	elastase, neutrophil expressed(ELANE)
TNMD	Homo sapiens	tenomodulin(TNMD)
CD160	Homo sapiens	CD160 molecule(CD160)
PSG3	Homo sapiens	pregnancy specific beta-1-glycoprotein 3(PSG3)
LRRC26	Homo sapiens	leucine rich repeat containing 26(LRRC26)
SLC26A3	Homo sapiens	solute carrier family 26 member 3(SLC26A3)
TLCD1	Homo sapiens	TLC domain containing 1(TLCD1)
PTGDR	Homo sapiens	prostaglandin D2 receptor(PTGDR)
GPA33	Homo sapiens	glycoprotein A33(GPA33)
TXK	Homo sapiens	TXK tyrosine kinase(TXK)
FCRLA	Homo sapiens	Fc receptor like A(FCRLA)
SCN11A	Homo sapiens	sodium voltage-gated channel alpha subunit 11(SCN11A)
AMY1B	Homo sapiens	amylase, alpha 1B (salivary)(AMY1B)

GPR182	Homo sapiens	G protein-coupled receptor 182(GPR182)
ALPI	Homo sapiens	alkaline phosphatase, intestinal(ALPI)
TRIM10	Homo sapiens	tripartite motif containing 10(TRIM10)
ALPP	Homo sapiens	alkaline phosphatase, placental(ALPP)
HK3	Homo sapiens	hexokinase 3(HK3)
KIR3DX1	Homo sapiens	killer cell immunoglobulin like receptor, three Ig domains X1(KIR3DX1)
NMU	Homo sapiens	neuromedin U(NMU)
ABCA12	Homo sapiens	ATP binding cassette subfamily A member 12(ABCA12)
SIM2	Homo sapiens	single-minded family bHLH transcription factor 2(SIM2)
MOGAT2	Homo sapiens	monoacylglycerol O-acyltransferase 2(MOGAT2)
HAPLN1	Homo sapiens	hyaluronan and proteoglycan link protein 1(HAPLN1)
HIST1H2BC	Homo sapiens	histone cluster 1 H2B family member c(HIST1H2BC)
PLA2G10	Homo sapiens	phospholipase A2 group X(PLA2G10)
FTCD	Homo sapiens	formimidoyltransferasecyclodeaminase(FTCD)
GJB5	Homo sapiens	gap junction protein beta 5(GJB5)
NPY1R	Homo sapiens	neuropeptide Y receptor Y1(NPY1R)
STAB2	Homo sapiens	stabilin 2(STAB2)
CCL15	Homo sapiens	C-C motif chemokine ligand 15(CCL15)
DKK4	Homo sapiens	dickkopf WNT signaling pathway inhibitor 4(DKK4)
MTTP	Homo sapiens	microsomal triglyceride transfer protein(MTTP)
TBATA	Homo sapiens	thymus, brain and testes associated(TBATA)
CD19	Homo sapiens	CD19 molecule(CD19)
COL19A1	Homo sapiens	collagen type XIX alpha 1 chain(COL19A1)
SAGE1	Homo sapiens	sarcoma antigen 1(SAGE1)
FOXE1	Homo sapiens	forkhead box E1(FOXE1)
TUBAL3	Homo sapiens	tubulin alpha like 3(TUBAL3)
ACSM5	Homo sapiens	acyl-CoA synthetase medium-chain family member 5(ACSM5)
NR5A1	Homo sapiens	nuclear receptor subfamily 5 group A member 1(NR5A1)
APOBEC1	Homo sapiens	apolipoprotein B mRNA editing enzyme catalytic subunit 1(APOBEC1)
LRTM1	Homo sapiens	leucine rich repeats and transmembrane domains 1(LRTM1)
NUP62CL	Homo sapiens	nucleoporin 62 C-terminal like(NUP62CL)
KRTAP4-1	Homo sapiens	keratin associated protein 4-1(KRTAP4-1)
ZIC2	Homo sapiens	Zic family member 2(ZIC2)
GP9	Homo sapiens	glycoprotein IX platelet(GP9)
APOA2	Homo sapiens	apolipoprotein A2(APOA2)
APOA1	Homo sapiens	apolipoprotein A1(APOA1)
GATA5	Homo sapiens	GATA binding protein 5(GATA5)
TDGF1	Homo sapiens	teratocarcinoma-derived growth factor 1(TDGF1)
SLC4A1	Homo sapiens	solute carrier family 4 member 1 (Diego blood group)(SLC4A1)
SPATA21	Homo sapiens	spermatogenesis associated 21(SPATA21)

TMPRSS15	Homo sapiens	transmembrane protease, serine 15(TMPRSS15)
BEND4	Homo sapiens	BEN domain containing 4(BEND4)
STAP1	Homo sapiens	signal transducing adaptor family member 1(STAP1)
PNCK	Homo sapiens	pregnancy up-regulated nonubiquitousCaM kinase(PNCK)
TRIM40	Homo sapiens	tripartite motif containing 40(TRIM40)
GTSF1L	Homo sapiens	gametocyte specific factor 1 like(GTSF1L)
AJAP1	Homo sapiens	adherens junctions associated protein 1(AJAP1)
SP140	Homo sapiens	SP140 nuclear body protein(SP140)
KCNT1	Homo sapiens	potassium sodium-activated channel subfamily T member 1(KCNT1)
UPK3BL	Homo sapiens	uroplakin 3B-like(UPK3BL)
DEFA4	Homo sapiens	defensin alpha 4(DEFA4)
CD300LG	Homo sapiens	CD300 molecule like family member g(CD300LG)
DEFA3	Homo sapiens	defensin alpha 3(DEFA3)
KIR2DL1	Homo sapiens	killer cell immunoglobulin like receptor, two Ig domains and long cytoplasmic tail 1(KIR2DL1)
PAEP	Homo sapiens	progestagen associated endometrial protein(PAEP)
KIR2DL3	Homo sapiens	killer cell immunoglobulin like receptor, two Ig domains and long cytoplasmic tail 3(KIR2DL3)
CD300LB	Homo sapiens	CD300 molecule like family member b(CD300LB)
KIR3DL1	Homo sapiens	killer cell immunoglobulin like receptor, three Ig domains and long cytoplasmic tail 1(KIR3DL1)
TM4SF19	Homo sapiens	transmembrane 4 L six family member 19(TM4SF19)
BLK	Homo sapiens	BLK proto-oncogene, Src family tyrosine kinase(BLK)
TMEM217	Homo sapiens	transmembrane protein 217(TMEM217)
ITLN2	Homo sapiens	intelectin 2(ITLN2)
TMEM82	Homo sapiens	transmembrane protein 82(TMEM82)
KLRK1	Homo sapiens	killer cell lectin like receptor K1(KLRK1)
TCL1A	Homo sapiens	T-cell leukemia/lymphoma 1A(TCL1A)
KMO	Homo sapiens	kynurenine 3-monooxygenase(KMO)
OR2L13	Homo sapiens	olfactory receptor family 2 subfamily L member 13(OR2L13)
OVOL1	Homo sapiens	ovo like transcriptional repressor 1(OVOL1)
ENTPD8	Homo sapiens	ectonucleoside triphosphate diphosphohydrolase 8(ENTPD8)
CNR2	Homo sapiens	cannabinoid receptor 2(CNR2)
SH3TC2	Homo sapiens	SH3 domain and tetratricopeptide repeats 2(SH3TC2)
UPK2	Homo sapiens	uroplakin 2(UPK2)
LMAN1L	Homo sapiens	lectin, mannose binding 1 like(LMAN1L)
TLR10	Homo sapiens	toll like receptor 10(TLR10)
RFX4	Homo sapiens	regulatory factor X4(RFX4)
PRSS57	Homo sapiens	protease, serine 57(PRSS57)
HRASLS2	Homo sapiens	HRAS like suppressor 2(HRASLS2)
DRC1	Homo sapiens	dynein regulatory complex subunit 1(DRC1)
TNFRSF13B	Homo sapiens	TNF receptor superfamily member 13B(TNFRSF13B)

TNFRSF13C	Homo sapiens	TNF receptor superfamily member 13C(TNFRSF13C)
BFSP2	Homo sapiens	beaded filament structural protein 2(BFSP2)
SLC6A19	Homo sapiens	solute carrier family 6 member 19(SLC6A19)
HIST2H3C	Homo sapiens	histone cluster 2 H3 family member c(HIST2H3C)
AIM2	Homo sapiens	absent in melanoma 2(AIM2)
SALL3	Homo sapiens	spalt like transcription factor 3(SALL3)
P2RX5	Homo sapiens	purinergic receptor P2X 5(P2RX5)
HEATR9	Homo sapiens	HEAT repeat containing 9(HEATR9)
ZIC5	Homo sapiens	Zic family member 5(ZIC5)
OR6K3	Homo sapiens	olfactory receptor family 6 subfamily K member 3(OR6K3)
CD79B	Homo sapiens	CD79b molecule(CD79B)
ITGAD	Homo sapiens	integrin subunit alpha D(ITGAD)
NKG7	Homo sapiens	natural killer cell granule protein 7(NKG7)
SLC5A10	Homo sapiens	solute carrier family 5 member 10(SLC5A10)
FABP6	Homo sapiens	fatty acid binding protein 6(FABP6)
PPP1R14D	Homo sapiens	protein phosphatase 1 regulatory inhibitor subunit 14D(PPP1R14D)
INSL4	Homo sapiens	insulin like 4(INSL4)
PAX5	Homo sapiens	paired box 5(PAX5)
KIF2B	Homo sapiens	kinesin family member 2B(KIF2B)
CLEC17A	Homo sapiens	C-type lectin domain family 17 member A(CLEC17A)
AKR1C4	Homo sapiens	aldo-keto reductase family 1 member C4(AKR1C4)
ALAS2	Homo sapiens	5'-aminolevulinate synthase 2(ALAS2)
CXCR5	Homo sapiens	C-X-C motif chemokine receptor 5(CXCR5)
TPO	Homo sapiens	thyroid peroxidase(TPO)
NLRP4	Homo sapiens	NLR family pyrin domain containing 4(NLRP4)
GPR18	Homo sapiens	G protein-coupled receptor 18(GPR18)
OTX1	Homo sapiens	orthodenticle homeobox 1(OTX1)
SIGLEC11	Homo sapiens	sialic acid binding Ig like lectin 11(SIGLEC11)
SIGLEC14	Homo sapiens	sialic acid binding Ig like lectin 14(SIGLEC14)
UGT1A1	Homo sapiens	UDP glucuronosyltransferase family 1 member A1(UGT1A1)
NCR1	Homo sapiens	natural cytotoxicity triggering receptor 1(NCR1)
NCR3	Homo sapiens	natural cytotoxicity triggering receptor 3(NCR3)
HOXC11	Homo sapiens	homeobox C11(HOXC11)
CLECL1	Homo sapiens	C-type lectin like 1(CLECL1)
HOXC13	Homo sapiens	homeobox C13(HOXC13)
FRMD4A	Homo sapiens	FERM domain containing 4A(FRMD4A)
SIGLEC7	Homo sapiens	sialic acid binding Ig like lectin 7(SIGLEC7)
KLHL14	Homo sapiens	kelch like family member 14(KLHL14)
UGT2B11	Homo sapiens	UDP glucuronosyltransferase family 2 member B11(UGT2B11)
HBG1	Homo sapiens	hemoglobin subunit gamma 1(HBG1)
LCT	Homo sapiens	lactase(LCT)
UBE2T	Homo sapiens	ubiquitin conjugating enzyme E2 T(UBE2T)
IRX6	Homo sapiens	iroquois homeobox 6(IRX6)

CCM2L	Homo sapiens	CCM2 like scaffolding protein(CCM2L)
VPREB3	Homo sapiens	pre-B lymphocyte 3(VPREB3)
HBM	Homo sapiens	hemoglobin subunit mu(HBM)
IRX1	Homo sapiens	iroquois homeobox 1(IRX1)
TMEM40	Homo sapiens	transmembrane protein 40(TMEM40)
TDRD15	Homo sapiens	tudor domain containing 15(TDRD15)
C10orf90	Homo sapiens	chromosome 10 open reading frame 90(C10orf90)
SIGLEC9	Homo sapiens	sialic acid binding Ig like lectin 9(SIGLEC9)
TPSG1	Homo sapiens	tryptase gamma 1(TPSG1)
ARHGAP40	Homo sapiens	Rho GTPase activating protein 40(ARHGAP40)
PATL2	Homo sapiens	PAT1 homolog 2(PATL2)
PADI6	Homo sapiens	peptidyl arginine deiminase 6(PADI6)
SHISA8	Homo sapiens	shisa family member 8(SHISA8)
UGT1A8	Homo sapiens	UDP glucuronosyltransferase family 1 member A8(UGT1A8)
RASGRP2	Homo sapiens	RAS guanyl releasing protein 2(RASGRP2)
LEFTY2	Homo sapiens	left-right determination factor 2(LEFTY2)
GPSM2	Homo sapiens	G-protein signaling modulator 2(GPSM2)
HRG	Homo sapiens	histidine rich glycoprotein(HRG)
KRT4	Homo sapiens	keratin 4(KRT4)
PGA5	Homo sapiens	pepsinogen 5, group I (pepsinogen A)(PGA5)
FGFBP2	Homo sapiens	fibroblast growth factor binding protein 2(FGFBP2)
ZC3H12D	Homo sapiens	zinc finger CCCH-type containing 12D(ZC3H12D)
AMHR2	Homo sapiens	anti-Mullerian hormone receptor type 2(AMHR2)
COL4A3	Homo sapiens	collagen type IV alpha 3 chain(COL4A3)
CR1	Homo sapiens	complement C3b/C4b receptor 1 (Knops blood group)(CR1)
CNTD2	Homo sapiens	cyclin N-terminal domain containing 2(CNTD2)
NOX5	Homo sapiens	NADPH oxidase 5(NOX5)
SLC12A3	Homo sapiens	solute carrier family 12 member 3(SLC12A3)
FAM9C	Homo sapiens	family with sequence similarity 9 member C(FAM9C)
CNNM1	Homo sapiens	cyclin and CBS domain divalent metal cation transport mediator 1(CNNM1)
GDPD2	Homo sapiens	glycerophosphodiester phosphodiesterase domain containing 2(GDPD2)
LEP	Homo sapiens	leptin(LEP)
KRT73	Homo sapiens	keratin 73(KRT73)
SLCO1B3	Homo sapiens	solute carrier organic anion transporter family member 1B3(SLCO1B3)
KRT72	Homo sapiens	keratin 72(KRT72)
KRT75	Homo sapiens	keratin 75(KRT75)
GLYAT	Homo sapiens	glycine-N-acyltransferase(GLYAT)
NTS	Homo sapiens	neurotensin(NTS)
KRT79	Homo sapiens	keratin 79(KRT79)
MEP1A	Homo sapiens	meprin A subunit alpha(MEP1A)
DSC3	Homo sapiens	desmocollin 3(DSC3)
BANK1	Homo sapiens	B-cell scaffold protein with ankyrin repeats

		1(BANK1)
C11orf21	Homo sapiens	chromosome 11 open reading frame 21(C11orf21)
SCN4A	Homo sapiens	sodium voltage-gated channel alpha subunit 4(SCN4A)

Table S4.MI score and significant

moduels	Score	p-value
module12	0.026124605	4.27E-51
module7	0.017933423	3.93E-26
module6	0.00626524	2.58E-18
module10	0.006455416	3.47E-18
module2	0.008149956	1.16E-17
module5	0.007219576	2.10E-16
module9	0.016549361	4.20E-13
module1	0.017028928	9.62E-11
module3	0.016355257	8.91E-09
module8	0.010349142	4.36E-06
module11	0.008952165	0.000204452
module4	0.01250513	0.240569019

Table S5. Multivariate Cox regression for OS in the training dataset

	beta	HR (95%_CI_for_HR)	p.value
gender	-0.408	1.5043 (0.428-1.031)	0.06847
age	0.006	1.27 (0.76-2.05)	0.3659
Stage	1	1.2764 (10.39-4.119)	6.83E-01
3-PPI-Mod	0.70631	2.0265 (1.1954-3.436)	0.00873
